# Factors correlated with targeted prevention for prediabetes classified by impaired fasting glucose, impaired glucose tolerance, and elevated HbA1c: A population-based longitudinal study

**DOI:** 10.3389/fendo.2022.965890

**Published:** 2022-08-22

**Authors:** Xiaoyue Zhu, Zhipeng Yang, Zhiliang He, Jingyao Hu, Tianxiu Yin, Hexiang Bai, Ruoyu Li, Le Cai, Haijian Guo, Mingma Li, Tao Yan, You Li, Chenye Shen, Kaicheng Sun, Yu Liu, Zilin Sun, Bei Wang

**Affiliations:** ^1^ Key Laboratory of Environment Medicine and Engineering of Ministry of Education, Department of Epidemiology and Health Statistics, School of Public Health, Southeast University, Nanjing, China; ^2^ School of Software, Fudan University, Shanghai, China; ^3^ Integrated Business Management Office, Jiangsu Province Centre Disease Control and Prevention, Nanjing, China; ^4^ Yandu Centre for Disease Control and Prevention, Yancheng, China; ^5^ Jurong Centre for Disease Control and Prevention, Jurong, China; ^6^ Department of Endocrinology, Institute of Diabetes, Medical School, Southeast University, Nanjing, China

**Keywords:** prediabetes, prevention, correlated factors, obesity indicators, epidemiology

## Abstract

**Background:**

There is still controversy surrounding the precise characterization of prediabetic population. We aim to identify and examine factors of demographic, behavioral, clinical, and biochemical characteristics, and obesity indicators (anthropometric characteristics and anthropometric prediction equation) for prediabetes according to different definition criteria of the American Diabetes Association (ADA) in the Chinese population.

**Methods:**

A longitudinal study consisted of baseline survey and two follow-ups was conducted, and a pooled data were analyzed. Prediabetes was defined as either impaired fasting glucose (IFG), impaired glucose tolerance (IGT), or elevated glycosylated hemoglobin (HbA1c) according to the ADA criteria. Robust generalized estimating equation models were used.

**Results:**

A total of 5,713 (58.42%) observations were prediabetes (IGT, 38.07%; IGT, 26.51%; elevated HbA1c, 23.45%); 9.66% prediabetes fulfilled all the three ADA criteria. Among demographic characteristics, higher age was more evident in elevated HbA1c [adjusted OR (aOR)=2.85]. Female individuals were less likely to have IFG (aOR=0.70) and more likely to suffer from IGT than male individuals (aOR=1.41). Several inconsistency correlations of biochemical characteristics and obesity indicators were detected by prediabetes criteria. Body adiposity estimator exhibited strong association with prediabetes (D10: aOR=4.05). For IFG and elevated HbA1c, the odds of predicted lean body mass exceed other indicators (D10: aOR=3.34; aOR=3.64). For IGT, predicted percent fat presented the highest odds (D10: aOR=6.58).

**Conclusion:**

Some correlated factors of prediabetes under different criteria differed, and obesity indicators were easily measured for target identification. Our findings could be used for targeted intervention to optimize preventions to mitigate the obviously increased prevalence of diabetes.

## Introduction

Diabetes mellitus (DM), with major complications, is a burdensome and costly disease worldwide, in which type 2 diabetes mellitus (T2DM) accounts for approximately 90% of the total (1, 2). It is reported that 463 million people were living with DM in 2019, and the number is expected to increase to 700 million in 2045 globally (1). Meanwhile, the estimated global direct health expenditure on DM in 2019 is USD 760 billion, let alone its acute and long-term complications on health expenditures (2). Remarkably, China and India are the top 2 epicenters of the global epidemic of T2DM (3). Epidemiological study has revealed that approximately 11% of the Chinese were suffering from DM, with a significant proportion undiagnosed (4). Although China has committed to combat DM through health system reform and national initiatives, the burdens of DM and its complications remain an ongoing challenge (5–7).

People at intermediate stages of hyperglycemia conditions, which are considered as prediabetes, could be at high risk of developing DM, since a large proportion of people with prediabetes progresses towards DM (8). Despite the tremendous efforts for prolonging lives of DM patients, DM remains as the eighth leading cause of death among both sexes and fifth leading cause of death in women (9). Without doubt, it is more important to put focus on prediabetes, which can be the effective target of high-risk groups. As the vast majority of individuals with prediabetes are unaware of their diagnosis, it is vital that the associated conditions should be identified and people with high risk of prediabetes need to be targeted, so they may benefit from early intervention (10, 11). Moreover, prediabetes is a risk factor for the development of cardiovascular disease and stroke (12). Macrovascular complications may already present before the official diagnosis of DM; as a result, prediabetes should not be reflected as benign with the absence of co-morbid conditions (8, 10). Thus, target identification and early prevention for prediabetes are not only beneficial for the alarming epidemic of DM but also contribute to slow down the overall prevalence of diabetic complications (8, 12). A previous study has suggested that even though interventions in people classified as having prediabetes by screening have some efficacy in preventing or delaying onsets of DM in trial populations, “screen and treat” policies alone are unlikely to have substantial impact on the worsening epidemic of DM (13). Hence, effective strategies to identify, inform, and motivate individuals at risk to get tested, and appropriate managements to initiate lifestyle interventions to these people as well, are core components to assure widespread adoption of interventions at reasonable costs (14).

To halt the epidemic of diabetes, we need to target those at risk of developing prediabetes and steer effective strategies, which premises from understanding the characteristics of individuals at “high-risk” systematically. Even though the new thresholds for defining prediabetes have been around for more than 10 years, there is still controversy surrounding the precise characterization of prediabetic population (15). To date, little is known about the prediabetic conditions, and some knowledge of prediabetes gaps still remains in China (16), especially with regard to the potentially different characteristics of prediabetic subgroups classified by impaired fasting glucose (IFG), impaired glucose tolerance (IGT), or elevated glycosylated hemoglobin (HbA1c), in which more tailored and adjusted advices for interventions could be needed. As IFG, IGT, and elevated HbA1c pose different underlying pathophysiologies, the efficacy of different preventive measures may differ for these prediabetic groups (8). Thus, we investigated the overlap in populations that have prediabetes defined by one of the three prediabetes criteria. Furthermore, we aim to comprehensively identify and examine factors of demographic, behavioral, clinical, and biochemical characteristics, and obesity indicators (anthropometric characteristics and anthropometric prediction equation) that are correlated with prediabetes defined by different prediabetes criteria in the Chinese population.

## Materials and methods

### Study population and study design

**Figure 1:**
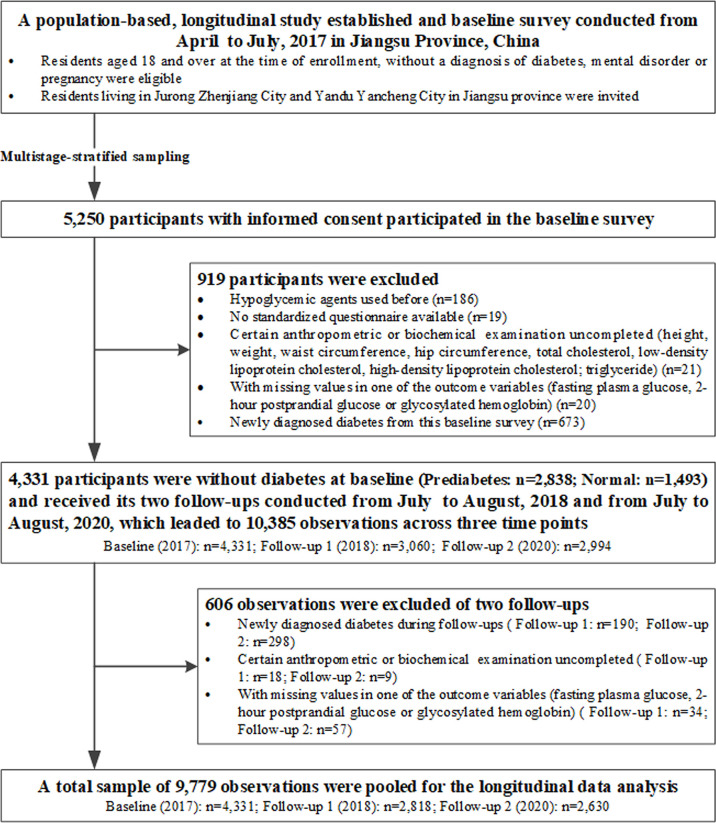
Flowchart for selection of study participants.

A longitudinal study, which consisted of a baseline survey conducted from April to July 2017 and its two follow-ups from July to August 2018 and from July to August 2020, was established in Jiangsu province, China. Multistage-stratified sampling method was used from Jurong Zhengjiang and Yandu Yancheng, two cities in Jiangsu province (**Supplementary Material 1: Figure S1**). Detailed information about study design, organization, and implementation of this longitudinal study has been described previously (17). Overall, 5,250 residents participated in the baseline survey, which consisted of a questionnaire and physical and laboratory examinations. A total of 4,331 participants without diabetes who met the inclusion criteria were recruited in the baseline study and received two follow-up studies: follow-up 1 (2018, n=3,060) and follow-up 2 (2020, n=2,994). We pooled data from these three studies with a total of 10,385 observations. As the aim of our study was putting focus on preventive efforts of people with high risk of diabetes, observations with newly diagnosed diabetes were excluded from the analysis sample (n=488). We further excluded those observations with certain anthropometric or biochemical examination uncompleted (n=27) and with missing values in one of the outcome variables (fasting plasma glucose (FPG), namely, 2-h postprandial glucose (2hPG) or HbA1c (n=91). This resulted in a final analysis sample of 9,779 observations across three time points. Thus, we obtained an analysis dataset with repeated observations, including 859 participates with one observation, 1,496 participants with two observations, and 1,976 participants with three observations. By design, **Figure 1** shows detailed information of the study flowchart, in which enrollment and exclusion criteria are presented.

This study was conducted in accordance with the Declaration of Helsinki. The study was viewed and approved by the Ethics Review Committee of Zhongda Hospital, Southeast University, and Jiangsu Provincial Centre for Disease Control and Prevention (JSJK2017-B003-02). All study participants provided written informed consent.

### Data collection and operational definitions

In all these three studies, participants completed an interview conducted by experienced local health workers using a standardized questionnaire to collect information regarding demographic characteristics (age, gender, education level and self-reported drug history, equivalent household income, and family history of diabetes), and behavioral characteristics (smoking status, drinking status, and regular exercise). These characteristics were further categorized as follows: age (<50/50–59/≥60), gender (male/female), education level (junior high school or below/senior high school or above), equivalent household income (low: per capita monthly household income < 1,000 RMB; moderate: 1,000 RMB ≤ per capita monthly household income < 3,000 RMB; high: per capita monthly household income ≥ 3000RMB), drug history (no/yes: drugs included antihypertensive agent, antilipemic agent, antithrombotic agent, antineoplastic agent, and non-steroid anti-inflammatory drug), and family history of diabetes (no/yes/unclear). Smoking status was divided as follows: non-smoker, current smoker or ex-smoker, and drinking status as (never/ever). Regular exercise was grouped as follows: no and yes (recreational sports activities more than 30 min per time, more than three times a week). Participants were asked to have at least a 5-min rest in a seated position before their systolic and diastolic blood pressures were measured by electronic sphygmomanometers. Hypertension was defined as a systolic blood pressure ≥140 mmHg and/or a diastolic blood pressure ≥90 mm Hg, or using antihypertensive agent (18).

Forearm venous blood samples were collected and measured for biochemical characteristics after overnight fasting for more than 8 h before examination. Subsequently, standard oral glucose tolerance tests (75 g anhydrous glucose) were administered and conducted without eating breakfast; 2 h later, a second blood sample was taken for 2hPG. Blood tests were all conducted in the same central laboratory (Adicon, Nanjing China) to maintain consistency and included FPG, 2hPG, HbA1c, total cholesterol (TC), low-density lipoprotein cholesterol (LDL-C), high-density lipoprotein cholesterol (HDL-C), and triglyceride (TG). Classifications of cholesterol and triglyceride levels were based on the Chinese guideline for the management of dyslipidemia in adults, which was consistent with the National Lipid Association Recommendation (19, 20). They were grouped as follows: TC (above desirable: <5.2 mmol/L; borderline high: 5.2–6.1 mmol/L; high: ≥6.2 mmol/L), LDL-C (desirable: <2.6 mmol/L; above desirable: 2.6–3.3 mmol/L; borderline high: 3.4–4.0 mmol/L; high: ≥4.1mmol/L), HDL-C (normal: ≥1.0 mmol/L; low: <1.0 mmol/L), and TG (above desirable: <1.7 mmol/L; borderline high, 1.7–2.2 mmol/L; high: ≥2.3 mmol/L). Weight was measured with a digital scale to the nearest 0.1 kg with light clothes, and height was measured to the nearest 0.1 cm using a wall-mounted stadiometer. Waist circumference (WC) and hip circumference were measured using a tape with a metric scale under standardized procedures, with the participants in a standing position. Waist-to-height ratio (WHtR) (21), waist-to-hip ratio (WHR) (22), body mass index (BMI) (23), ponderal index (PI) (24), conicity index (25), relative fat mass (RFM) (26), abdominal volume index (AVI) (27), lipid accumulation product (LAP) (28), visceral adiposity index (VAI) (29), Chinese visceral adiposity index (CVAI) (30), body roundness index (BRI) (31), and body adiposity estimator (BAE) were calculated based on the formulas, respectively (**Supplementary Materials 2**) (32). We derived predicted lean body mass, fat mass, and percent fat (%) by using anthropometric prediction equations developed and validated by the National Health and Nutrition Examination Survey previously, which included 7,531 men and 6,534 women who underwent dual-energy X-ray absorptiometry-measured examination (33). Details on the calculation of anthropometric characteristics and prediction equations can be found in **Supplementary Materials 2**. The thresholds for WC were specified according to the International Diabetes Foundation for the Chinese populations (WC > 80 cm for women and WC > 90 cm for men) (34). WHtR ≥ 0.50 was considered as the optimal cutoff value in the Chinese adults (35). According to the World Health Organization, the cutoff values for WHR were defined (0.85 in women and 0.90 in men) (34). The classification of BMI was defined according to the criteria of the working group on obesity in China (23): underweight (BMI < 18.5 kg/m^2^), normal (18.5 ≤ BMI < 24.0 kg/m^2^), overweight (24.0 ≤ BMI < 28.0 kg/m^2^), and obesity (BMI ≥ 28.0 kg/m^2^). We created quartiles for other anthropometric characteristics and anthropometric prediction equation, as there were lack of specific cutoffs for clinical practice (36), with quartile 1 (Q1) representing the lowest level and quartile 4 (Q4) standing for the highest level. All the factors of anthropometric characteristics and anthropometric prediction equation, considered as obesity indicators, were furtherly classified into deciles, in which decile 1 (D1) presented the lowest level and decile 10 (D10) showed the highest level. The method of questionnaires and physical and laboratory examinations for the two follow-ups was exactly as same as for the baseline survey. All variables, including demographic, behavioral, clinical and biochemical, and anthropometric characteristics, and anthropometric prediction equation were similarly assessed at each follow-up. Therefore, pooling of these three studies was allowed.

### Definitions of diabetes and prediabetes

Prediabetes and diabetes were defined according to the diagnostic criteria of the American Diabetes Association (ADA) (37). An FPG ≥ 7.0mmol/L and/or a 2hPG ≥ 11.1 mmol/L and/or an HbA1c ≥ 6.5% were defined as diabetes (37, 38). Participants with IFG (5.6 mmol/L ≤ FPG < 7.0 mmol/L) and/or IGT (7.8 mmol/L ≤ 2hPG < 11.1 mmol/L) and/or elevated HbA1c (5.7% ≤ HbA1c < 6.5%) were defined as prediabetes (37). Accordingly, people with an FPG <5.6 mmol/L and 2hPG <7.8 mmol/L and HbA1c 5.7% were defined as normal glucose tolerance.

### Statistical analysis

Descriptive statistics were used to present participant characteristics, with mean ± standard deviations (SDs), median with inter quartile range, or numbers with percentages to present characteristics of participants when appropriate. The *χ* ^2^ test for categorical variables or *t* test and the Wilcoxon tests for continuous variables were introduced for statistical evaluations of demographic, behavioral, clinical, and biochemical characteristics, and obesity indicators (anthropometric characteristics and anthropometric prediction equation) between prediabetes and the normal group at baseline. Given the correlations between repetitive observations among baseline study and follow-up studies, which violated independence assumptions that are required for traditional regression procedures (39), and in order to increase the analytical power, robust generalized estimating equation (GEE) models with a binary distribution using a logit link and exchange structure were applied for the pooled analysis sample. The correlation coefficients between two repetitive outcome measurements at each follow-up times were approximately equal (**Supplementary Materials 1: Table S1**). Univariate and multivariate models were simultaneously adopted with odd ratios (ORs) and 95% confidence intervals (CIs) to analyze factors correlated with prediabetes classified by the three diagnostic criteria separately and combined. For the aspects of anthropometric characteristics and anthropometric prediction equation, obesity indicators were correlated with each other and were evaluated in separate multivariate models. Covariate missingness in our pooled sample ranged from 0% to 0.34% (0.05% for drinking status and 0.34% for hypertension). We created five imputed datasets using multivariate imputation by chained equations (MICE) package in R software, and the main analysis results were pooled after appropriate transformation (40). All statistical analyses were performed with R software (version 4.1.1). Two-sided *p*-values <0.05 were considered as statistically significant.

## Results

### Baseline and the total observation characteristics

A total of 4,331 participants without diabetes (prediabetes: n=2,838; normal: n=1,493) were included in the baseline study in 2017 and received its two follow-ups in 2018 and 2020, respectively. Hence, a total sample of 9,779 observations that fitted all inclusion criteria (baseline: n=4,331; follow-up 1 = 2,818; follow-up 2 = 2,630) were pooled for the longitudinal data analysis (**Figure 1**). A total of 1,672 (38.61%) men and 2,659 (61.39%) women were included at baseline. The average ages were 52.03 ± 8.61 for prediabetes and 48.64 ± 9.97 for the normal group at study entry. All of the characteristics were compared between the prediabetes and normal group for baseline study (**Supplementary Materials 1: Table S2**). A summary on the characteristics of observations combined by three studies with and without prediabetes is presented in **Table 1**. Totally, 5,713 (58.42%) observations were prediabetes (IGT: 38.07%; IGT: 26.51%; elevated HbA1c: 23.45%).

**Table 1:** Characteristics of the observations without diabetes.

	Criterions of prediabetes					
Variables	IFG (n=3,723)	IGT (n=2,592)	Elevated HbA1c (n=2,293)	Prediabetes^a^ (n=5,713)	Normal^b^ (n=4,066)	Total (n=9,779)
**Studies, n (%)**						
**Baseline (2017)**	2,305 (61.91)	1,140 (43.98)	901 (39.29)	2,838 (49.68)	1,493 (36.72)	4,331
Follow-up 1 (2018)	828 (22.24)	723 (27.89)	424 (18.49)	1,369 (23.96)	1,449 (35.63)	2,818
Follow-up 2 (2020)	590 (15.85)	729 (28.12)	968 (42.22)	1,506 (26.36)	1,124 (27.64)	2,630
**Demographic characteristics**						
**Age (y), n (%)**						
<50	1,081 (29.04)	693 (26.74)	439 (19.14)	1,586 (27.76)	1,625 (39.97)	3,211
50–59	1,595 (42.84)	1,075 (41.47)	1,091 (47.58)	2,466 (43.17)	1,615 (39.72)	4,081
≥60	1,047 (28.12)	824 (31.79)	763 (33.28)	1661 (29.07)	826 (20.31)	2,487
**Gender, n (%)**						
Male	1,628 (43.73)	905 (34.92)	852 (37.16)	2,283 (39.96)	1,386 (34.09)	3,669
Female	2,095 (56.27)	1,687 (65.08)	1,441 (62.84)	3,430 (60.04)	2,680 (65.91)	6,110
**Education level, n (%)**						
Junior high school or below	3,178 (85.36)	2,244 (86.57)	1,998 (87.13)	4,910 (85.94)	3,376 (83.03)	8,286
Senior high school or above	545 (14.64)	348 (13.43)	295 (12.87)	803 (14.06)	690 (16.97)	1493
**Equivalent household income**						
Low	1,243 (33.39)	879 (33.91)	797 (34.76)	1,907 (33.38)	1,214 (29.86)	3,121
Moderate	1,358 (36.48)	994 (38.35)	869 (37.90)	2,129 (37.27)	1,448 (35.61)	3,577
High	1,122 (30.14)	719 (27.74)	627 (27.34)	1,677 (29.35)	1,404 (34.53)	3,081
**Drug history, n (%)**						
No	2,554 (68.60)	1,689 (65.16)	1,512 (65.94)	3,951 (69.16)	3,266 (80.32)	7,217
Yes	1,169 (31.40)	903 (34.84)	781 (34.06)	1,762 (30.84)	800 (19.68)	2,562
**Family history of diabetes, n (%)**						
No	2,926 (78.59)	1,967 (75.89)	1,776 (77.45)	4,461 (78.08)	3,245 (79.81)	7,706
Yes	654 (17.57)	491 (18.94)	439 (19.15)	1,010 (17.68)	646 (15.89)	1,656
Unclear	143 (3.84)	134 (5.17)	78 (3.40)	242 (4.24)	175 (4.30)	417
**Behavioral characteristics**						
**Smoking status, n (%)**						
Non-smoker	2,758 (74.08)	2,031 (78.36)	1,750 (76.32)	4,339 (75.95)	3,248 (79.88)	7,587
Current smoker	787 (21.14)	455 (17.55)	449 (19.58)	1,135 (19.87)	723 (17.78)	1,858
Ex-smoker	178 (4.78)	106 (4.09)	94 (4.10)	239 (4.18)	95 (2.34)	334
**Drinking status, n (%) ** ^c^						
Ever	1,112 (29.90)	640 (24.71)	533 (23.24)	1,522 (26.66)	817 (20.10)	2,339
Never	2,607 (70.10)	1,950 (75.29)	1,760 (76.76)	4,187 (73.34)	3,248 (79.90)	7,435
**Regular exercise, n (%)**						
Yes	1,438 (38.62)	1,096 (42.28)	842 (36.72)	2,252 (39.42)	1,869 (45.97)	4,121
No	2,285 (61.38)	1,496 (57.72)	1,451 (63.28)	3,461 (60.58)	2,197 (54.03)	5,658
**Clinical and biochemical characteristics**						
**Hypertension, n (%) ** ^d^						
No	2,178 (58.63)	1,421 (54.89)	1,287 (56.27)	3,377 (59.25)	2,981 (73.68)	6,358
Yes	1,537 (41.37)	1,168 (45.11)	1,000 (43.73)	2,323 (40.75)	1,065 (26.32)	3,388
**TC (mmol/L), n (%)**						
Above desirable (<5.2)	2,522 (67.74)	1,735 (66.94)	1,501 (65.46)	3,936 (68.90)	3,282 (80.72)	7,218
Borderline high (5.2–6.1)	945 (25.38)	662 (25.54)	609 (26.56)	1,386 (24.26)	661 (16.26)	2,047
High (≥6.2)	256 (6.88)	195 (7.52)	183 (7.98)	391 (6.84)	123 (3.02)	514
**LDL-C (mmol/L), n (%)**						
Desirable (<2.6)	1,837 (49.34)	1,227 (47.34)	1,028 (44.83)	2,837 (49.66)	2,493 (61.31)	5,330
Above desirable (2.6–3.3)	1,398 (37.55)	1,010 (38.97)	904 (39.42)	2,149 (37.61)	1,262 (31.04)	3,411
Borderline high (3.4–4.0)	395 (10.61)	292 (11.27)	294 (12.82)	590 (10.33)	258 (6.35)	848
High (≥4.1)	93 (2.50)	63 (2.43)	67 (2.92)	137 (2.40)	53 (1.30)	190
**HDL-C (mmol/L), n (%)**						
Normal (≥1.0)	3,500 (94.01)	2,416 (93.21)	2,176 (94.90)	5,373 (94.05)	3,849 (94.66)	9,222
Low (<1.0)	223 (5.99)	176 (6.79)	117 (5.10)	340 (5.95)	217 (5.34)	557
**TG (mmol/L), n (%)**						
Above desirable (<1.7)	2,272 (61.03)	1,428 (55.09)	1,459 (63.63)	3,524 (61.68)	3,011 (74.05)	6,535
Borderline high (1.7–2.2)	669 (17.97)	535 (20.64)	396 (17.27)	1,026 (17.96)	515 (12.67)	1,541
High (≥2.3)	782 (21.00)	629 (24.27)	438 (19.10)	1,163 (20.36)	540 (13.28)	1,703
**Anthropometric characteristics**						
**WC (cm), n (%)**						
Normal	1,759 (47.25)	1,029 (39.70)	1,056 (46.05)	2,653 (46.44)	2,261 (55.61)	4,914
Non-standard	1,964 (52.75)	1,563 (60.30)	1,237 (53.95)	3,060 (53.56)	1,805 (44.39)	4,865
**WHtR, n (%)**						
<0.5	1,032 (27.72)	580 (22.38)	675 (29.44)	1,598 (27.97)	1,565 (38.49)	3,163
≥0.5	2,691 (72.28)	2,012 (77.62)	1,618 (70.56)	4,115 (72.03)	2,501 (61.51)	6,616
**WHR, n (%)**						
Normal	1,169 (31.40)	665 (25.66)	706 (30.79)	1,799 (31.49)	1,725 (42.42)	3,524
Non-standard	2,554 (68.60)	1,927 (74.34)	1,587 (69.21)	3,914 (68.51)	2,341 (57.58)	6,255
**BMI (kg/m^2^), n (%)**						
Underweight (<18.5)	33 (0.89)	22 (0.85)	30 (1.31)	68 (1.19)	85 (2.09)	153
Normal (18.5–23.9)	1,184 (31.80)	732 (28.24)	729 (31.79)	1,863 (32.61)	1,795 (44.15)	3,658
Overweight (24.0–27.9)	1,686 (45.29)	1,145 (44.17)	1,018 (44.40)	2,555 (44.72)	1,645 (40.46)	4,200
Obesity (≥28.0)	820 (22.03)	693 (26.74)	516 (22.50)	1227 (21.48)	541 (13.30)	1,768
**PI (kg/m^3^), n (%)**						
Q1 (<14.12)	755 (20.28)	415 (16.01)	484 (21.11)	1,196 (20.94)	1,242 (30.55)	2,438
Q2 (14.12–15.21)	912 (24.50)	568 (21.91)	490 (21.37)	1,357 (23.75)	1,100 (27.05)	2,457
Q3 (15.52–17.05)	966 (25.94)	681 (26.28)	631 (27.52)	1,503 (26.31)	947 (23.29)	2,450
Q4 (≥17.06)	1,090 (29.28)	928 (35.80)	688 (30.00)	1,657 (29.00)	777 (19.11)	2,434
**CI (m^3/2^·kg^1/2^), n (%)**						
Q1 (<43.10)	728 (19.55)	464 (17.90)	509 (22.19)	1,196 (20.94)	1,268 (31.18)	2,464
Q2 (43.10–48.49)	864 (23.21)	578 (22.30)	547 (23.86)	1,353 (23.68)	1,044 (25.68)	2,397
Q3 (48.50–54.49)	1,011 (27.16)	697 (26.89)	576 (25.12)	1,501 (26.27)	956 (23.51)	2,457
Q4 (≥54.50)	1,120 (30.08)	853 (32.91)	661 (28.83)	1,663 (29.11)	798 (19.63)	2,461
**RFM, n (%)**						
Q1 (<27.04)	1,023 (27.48)	498 (19.21)	553 (24.12)	1,429 (25.01)	1,008 (24.79)	2,437
Q2 (27.04–34.17)	916 (24.60)	607 (23.42)	531 (23.16)	1,387 (24.28)	1,058 (26.02)	2,445
Q3 (34.18–39.00)	786 (21.11)	626 (24.15)	561 (24.46)	1,328 (23.25)	1,120 (27.55)	2,448
Q4 (≥39.00)	998 (26.81)	861 (33.22)	648 (28.26)	1,569 (27.46)	880 (21.64)	2,449
**AVI (L), n (%)**						
Q1 (<12.21)	755 (20.28)	467 (18.02)	538 (23.46)	1,221 (21.37)	1,224 (30.10)	2,445
Q2 (12.21–14.19)	867 (23.29)	603 (23.26)	535 (23.33)	1,360 (23.81)	1,058 (26.02)	2,418
Q3 (14.20–16.40)	999 (26.83)	691 (26.66)	582 (25.38)	1,490 (26.08)	981 (24.13)	2,471
Q4 (≥16.40)	1,102 (29.60)	831 (32.06)	638 (27.83)	1,642 (28.74)	803 (19.75)	2,445
**LAP (cm·mmol/L), n (%)**						
Q1 (<17.81)	772 (20.73)	387 (14.93)	502 (21.89)	1,185 (20.74)	1,258 (30.94)	2,443
Q2 (17.81–30.74)	854 (22.94)	530 (20.45)	527 (22.98)	1,314 (23.00)	1,132 (27.84)	2,446
Q3 (30.75–52.13)	988 (26.54)	727 (28.05)	616 (26.87)	1,530 (26.78)	913 (22.45)	2,443
Q4 (≥52.14)	1,109 (29.79)	948 (36.57)	648 (28.26)	1,684 (29.48)	763 (18.77)	2,447
**VAI, n (%)**						
Q1 (<0.95)	896 (24.06)	434 (16.74)	511 (22.28)	1,296 (22.69)	1149 (28.26)	2445
Q2 (0.95–1.46)	864 (23.21)	553 (21.34)	550 (23.99)	1,338 (23.42)	1107 (27.23)	2445
Q3 (1.47–2.35)	918 (24.66)	706 (27.24)	623 (27.17)	1,466 (25.66)	978 (24.05)	2444
Q4 (≥2.36)	1,045 (28.07)	899 (34.68)	609 (26.56)	1,613 (28.23)	832 (20.46)	2,445
**CVAI, n (%)**						
Q1 (<66.50)	751 (20.17)	374 (14.43)	415 (18.10)	1,115 (19.52)	1,331 (32.73)	2,446
Q2 (66.50–92.09)	868 (23.31)	556 (21.45)	546 (23.81)	1,366 (23.91)	1,072 (26.37)	2,438
Q3 (92.10–115.59)	963 (25.87)	727 (28.05)	618 (26.95)	1,511 (26.45)	937 (23.04)	2,448
Q4 (≥115.60)	1,141 (30.65)	935 (36.07)	714 (31.14)	1,721 (30.12)	726 (17.86)	2,447
**BRI, n (%)**						
Q1 (<3.12)	774 (20.79)	416 (16.05)	506 (22.07)	1,202 (21.04)	1,243 (30.57)	2,445
Q2 (3.12–3.84)	889 (23.88)	585 (22.57)	546 (23.81)	1,371 (24.00)	1,073 (26.39)	2,444
Q3 (3.85–4.60)	991 (26.62)	700 (27.00)	581 (25.34)	1,505 (26.34)	940 (23.12)	2,445
Q4 (≥4.61)	1,069 (28.71)	891 (34.38)	660 (28.78)	1,635 (28.62)	810 (19.92)	2,445
**BAE, n (%)**						
Q1 (<26.88)	974 (26.16)	464 (17.90)	480 (20.93)	1,363 (23.86)	1,082 (26.61)	2,445
Q2 (26.88–33.45)	928 (24.93)	600 (23.15)	532 (23.20)	1,385 (24.24)	1,057 (25.99)	2,442
Q3 (33.46–38.16)	789 (21.19)	621 (23.96)	571 (24.91)	1,343 (23.51)	1,103 (27.13)	2,446
Q4 (≥38.17)	1,032 (27.72)	907 (34.99)	710 (30.96)	1,622 (28.39)	824 (20.27)	2,446
**Anthropometric prediction equation ** ^e^						
**Lean body mass (kg), n (%)**						
Q1 (<34.70)	764 (20.52)	608 (23.45)	526 (22.94)	1,282 (22.44)	1,143 (28.11)	2,425
Q2 (34.70–38.79)	845 (22.70)	672 (25.93)	594 (25.91)	1,388 (24.30)	1,089 (26.78)	2,477
Q3 (38.80–47.89)	978 (26.27)	657 (25.35)	580 (25.29)	1,477 (25.85)	953 (23.44)	2,430
Q4 (≥47.90)	1,136 (30.51)	655 (25.27)	593 (25.86)	1,566 (27.41)	881 (21.67)	2,447
**Fat mass (kg), n (%)**						
Q1 (<17.80)	891 (23.93)	455 (17.55)	511 (22.29)	1,314 (23.00)	1,133 (27.87)	2,447
Q2 (17.80–21.75)	869 (23.35)	577 (22.26)	500 (21.81)	1,332 (23.31)	1,112 (27.35)	2,444
Q3 (21.76–25.70)	935 (25.11)	685 (26.43)	605 (26.38)	1,456 (25.49)	985 (24.22)	2,441
Q4 (≥25.71)	1,028 (27.61)	875 (33.76)	677 (29.52)	1,611 (28.20)	836 (20.56)	2,447
**Percent fat (%), n (%)**						
Q1 (<27.30)	1,053 (28.28)	522 (20.14)	568 (24.77)	1,460 (25.55)	982 (24.15)	2,442
Q2 (27.30–35.99)	876 (23.53)	568 (21.91)	473 (20.63)	1,328 (23.25)	1,118 (27.50)	2,446
Q3 (36.00–39.29)	777 (20.87)	598 (23.07)	553 (24.12)	1,313 (22.98)	1,137 (27.96)	2,450
Q4 (≥39.30)	1,017 (27.32)	904 (34.88)	699 (30.48)	1,612 (28.22)	829 (20.39)	2,441

Data were presented as number (percentage) for categorical variables.

a Prediabetes: IFG or IGT or elevated HbA1c.

b Normal: without IFG, IGT, or elevated HbA1c.

c Five missing value in this characteristic.

d Thirty-three missing value in this characteristic.

e Lean body mass, fat mass, and percent fat were derived from a validated anthropometric prediction equation.

IFG, impaired fasting glucose; IGT, impaired glucose tolerance; HbA1c, glycosylated hemoglobin; TC, total cholesterol; LDL-C, low-density lipoprotein cholesterol; HDL-C, high-density lipoprotein cholesterol; TG, triglyceride; IQR, interquartile range; WC, waist circumference; WHtR, waist-to-height ratio; WHR, waist-to-hip ratio; BMI, body mass index; PI, ponderal index; CI, conicity index; RFM, relative fat mass; AVI, abdominal volume index; LAP, lipid accumulation product; VAI, visceral adiposity index; CVAI, Chinese visceral adiposity index; BRI, body roundness index; BAE, body adiposity estimator.

### Overlap of prediabetes classified by three criteria

**Figure 2:**
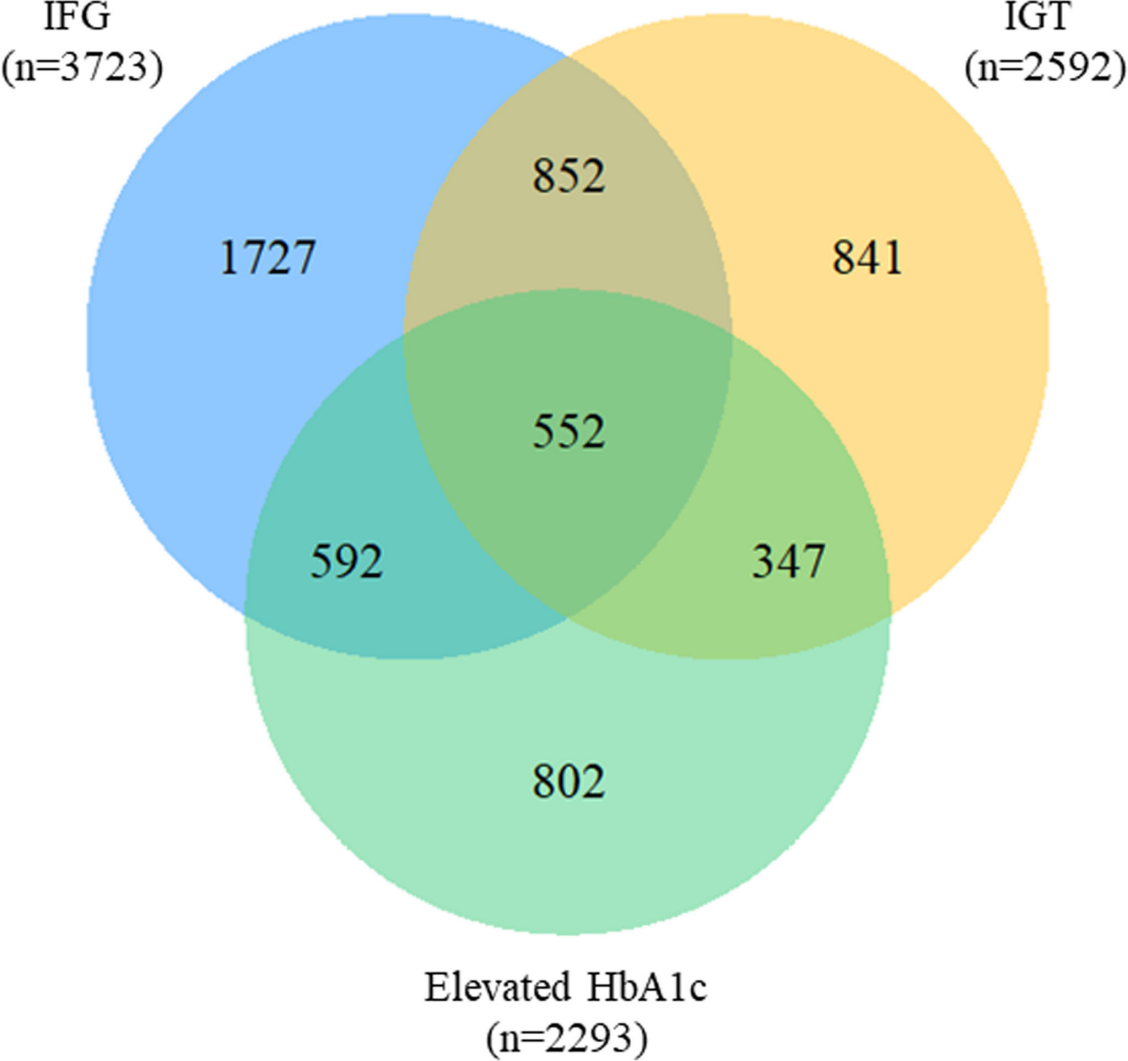
Venn diagram for the overlap of prediabetes criteria. IFG, impaired fasting glucose; IGT, impaired glucose tolerance; HbA1c, glycosylated hemoglobin.

A total of 552 (9.66%) observations satisfied all the three ADA criteria among 5,713 prediabetes, and 1,791(31.35%) fulfilled two of them simultaneously. A total of 852 (14.91%) overlapped between IFG and IGT, showing the largest overlap, whereas the smallest (6.07%) was found between IGT and elevated HbA1c (**Figure 2**). In the stratified analyses, the distributions were consistent with the total observations. Proportion of IFG were higher in men (71.31%) than in women (61.08%) with prediabetes, whereas the proportion of IGT or elevated HbA1c was higher in women (49.18%, 42.01%) than in men (39.64%, 37.32%). Additionally, the proportion of prediabetes fulfilling all the criteria was lower in men (8.63%) than in women (10.35%) (**Supplementary Materials 1: Figure S2**).

### Correlated factors for prediabetes

Compared with participants aged below 50 years, the higher age groups were more likely to suffer from prediabetes by multivariate analysis (*p*_trend_<0.01), especially in participants aged ≥ 60 years [adjusted OR (aOR)=1.74, 95% CI: 1.52–2.00]. Similar associations were also found in IGT (aOR=1.74, 95% CI: 1.50–2.01) and elevated HbA1c, which seemed more evident (aOR=2.85, 95% CI: 2.42–3.35). However, no significant association was detected between age groups and IFG (*p*_trend_=0.22). Being a woman (aOR=0.83, 95% CI: 0.72–0.96) and having a higher education level (aOR=0.80, 95% CI: 0.69-0.92) significantly decreased the likelihood of having prediabetes. According to the three criteria of prediabetes, women were less likely to have IFG and more likely to suffer from IGT than men. No significant association was found in gender for elevated HbA1c. Additionally, only IFG presented association with education levels. High equivalent household income was only correlated with IFG (aOR=0.88, 95% CI: 0.78–0.99). No significant associations of drug history were found in any prediabetes types. People with family history of diabetes consistently showed increased odds for having prediabetes of any criterion except for IFG (aOR=1.09, 95% CI: 0.95–1.24) (**Supplementary Materials 3: Table S1**). For behavioral characteristics, smoking status showed no association with prediabetes. Compared with people drinking alcohol, those who never drink showed decreased likelihood of having prediabetes (aOR=0.82, 95% CI: 0.72–0.93), while a contrary association was detected in elevated HbA1c category (aOR=1.19, 95% CI: 1.03–1.38). People without regular exercise presented higher odder (aOR=1.43, 95% CI: 1.32–1.55), but not for IGT (**Supplementary Materials 3: Table S1**). In terms of clinical and biochemical characteristics, having hypertension (aOR=1.33, 95% CI: 1.17–1.53), high TC (aOR=1.97, 95% CI: 1.49–2.62), above desirable LDL-C (aOR=1.18, 95% CI: 1.06–1.30), and high TG (aOR=1.35, 95% CI: 1.19–1.54) were associated with increased odds of prediabetes. Generally, factors of this aspect showed consistent associations categorized by prediabetes criteria, except the correlation between TC and elevated HbA1c (**Supplementary Materials 3: Table S1**).

**Figure 3:**
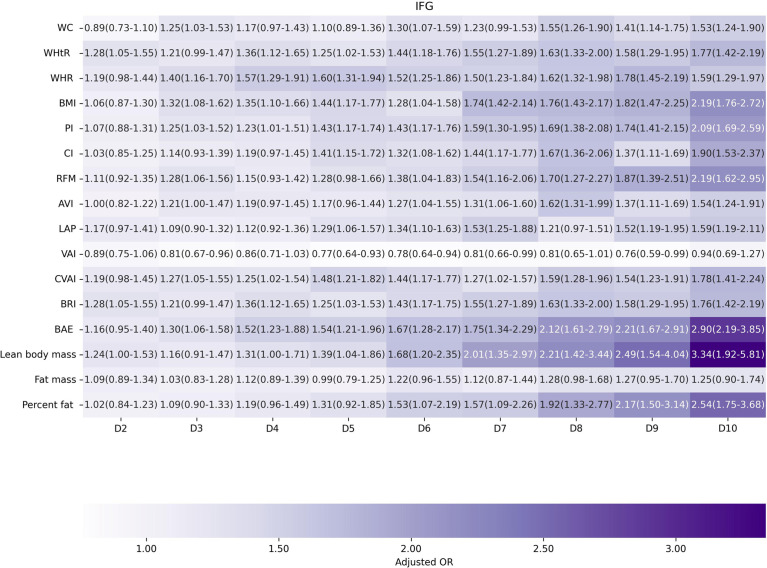
The association between deciles of obesity indicators and impaired fasting glucose. The first decile (D1) was used as reference. Adjusted for age, gender, education level, equivalent household income, drug history, family history of diabetes, smoking status, drinking status, regular exercise, hypertension, TC, LDL-C, HDL-C, and TG; for lean body mass, fat mass and height were further adjusted; for fat mass, lean body mass and height were further adjusted. WC, waist circumference; WHtR, waist-to-height ratio; WHR, waist-to-hip ratio; BMI, body mass index; PI, ponderal index; CI, conicity index; RFM, relative fat mass; AVI, abdominal volume index; LAP, lipid accumulation product; VAI, visceral adiposity index; CVAI, Chinese visceral adiposity index; BRI, body roundness index; BAE, body adiposity estimator.

**Figure 4:**
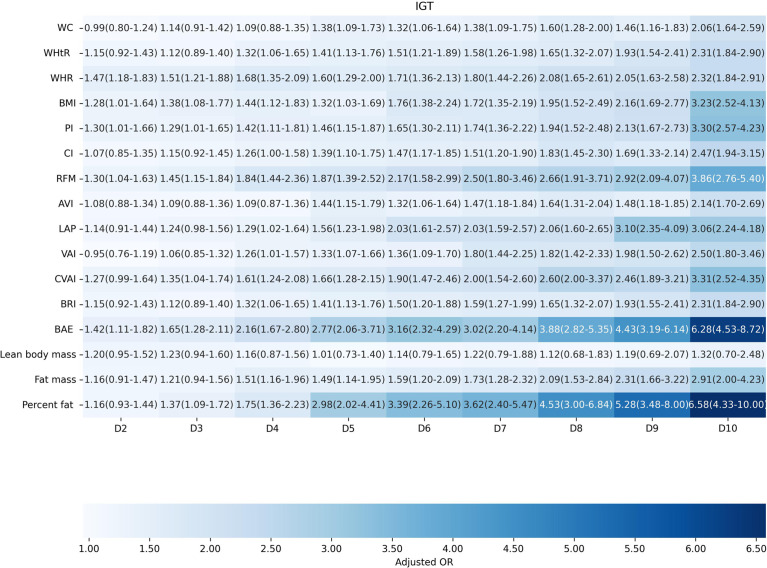
The association between deciles of obesity indicators and impaired glucose tolerance. The first decile (D1) was used as reference. Adjusted for age, gender, education level, equivalent household income, drug history, family history of diabetes, smoking status, drinking status, regular exercise, hypertension, TC, LDL-C, HDL-C, and TG; for lean body mass, fat mass and height were further adjusted; for fat mass, lean body mass and height were further adjusted. WC, waist circumference; WHtR, waist-to-height ratio; WHR, waist-to-hip ratio; BMI, body mass index; PI, ponderal index; CI, conicity index; RFM, relative fat mass; AVI, abdominal volume index; LAP, lipid accumulation product; VAI, visceral adiposity index; CVAI, Chinese visceral adiposity index; BRI, body roundness index; BAE, body adiposity estimator.

**Figure 5:**
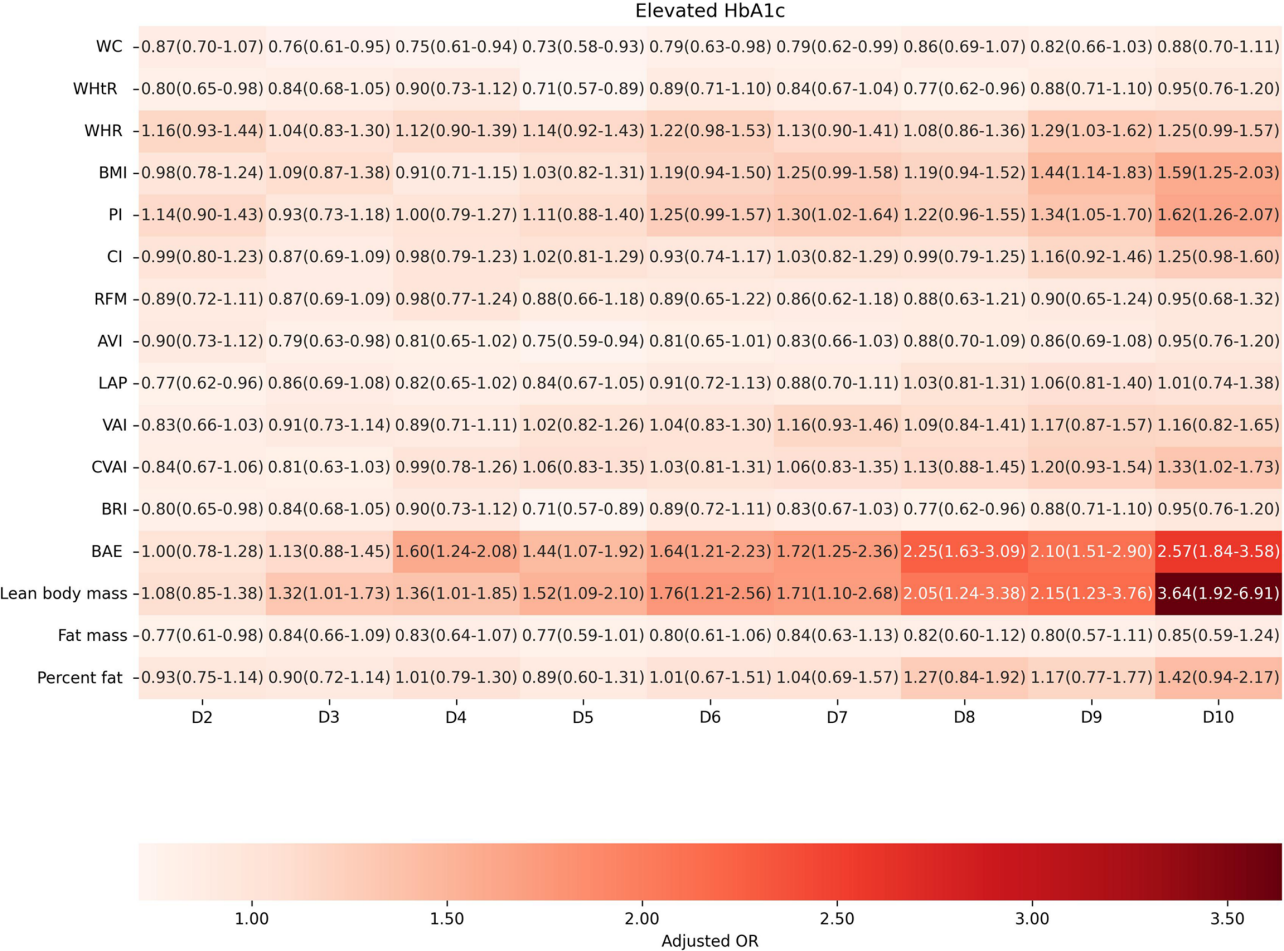
The association between deciles of obesity indicators and elevated HbA1c. The first decile (D1) was used as reference. Adjusted for age, gender, education level, equivalent household income, drug history, family history of diabetes, smoking status, drinking status, regular exercise, hypertension, TC, LDL-C, HDL-C, and TG; for lean body mass, fat mass and height were further adjusted; for fat mass, lean body mass and height were further adjusted. WC, waist circumference; WHtR, waist-to-height ratio; WHR, waist-to-hip ratio; BMI, body mass index; PI, ponderal index; CI, conicity index; RFM, relative fat mass; AVI, abdominal volume index; LAP, lipid accumulation product; VAI, visceral adiposity index; CVAI, Chinese visceral adiposity index; BRI, body roundness index; BAE, body adiposity estimator.

**Figure 6:**
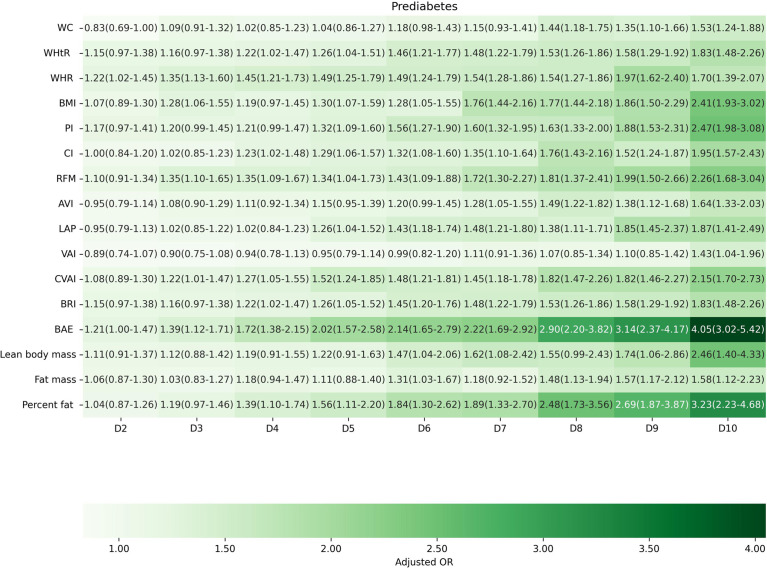
The association between deciles of obesity indicators and prediabetes based on the combined criteria of American Diabetes Association. The first decile (D1) was used as reference. Adjusted for age, gender, education level, equivalent household income, drug history, family history of diabetes, smoking status, drinking status, regular exercise, hypertension, TC, LDL-C, HDL-C, and TG; for lean body mass, fat mass and height were further adjusted; for fat mass, lean body mass and height were further adjusted. WC, waist circumference; WHtR, waist-to-height ratio; WHR, waist-to-hip ratio; BMI, body mass index; PI, ponderal index; CI, conicity index; RFM, relative fat mass; AVI, abdominal volume index; LAP, lipid accumulation product; VAI, visceral adiposity index; CVAI, Chinese visceral adiposity index; BRI, body roundness index; BAE, body adiposity estimator.

Factors of anthropometric characteristics and anthropometric prediction equation were also presented. Among these 16 obesity indicators, BAE was correlated with all classifications of prediabetes, and only VAI was not significantly associated with prediabetes classified by any criterion in multivariate models. Non-standard WC, WHtR, WHR, and higher levels of BMI were correlated with increased odds of prediabetes. These correlations were consistent according to prediabetes diagnostic criteria, except for HbA1c. For other obesity indicators, people with higher quartiles had higher odds of experiencing prediabetes. Inconsistencies were found in CI, RFM, AVI, LAP, CVAI, BRI, predicted fat mass, and percent fat classified by prediabetes criteria (**Supplementary Materials 3: Table S1**). We further introduced deciles of these obesity indicators to examine the associations in finer categories (**Supplementary Materials 1: Table S3**; **Figures 3**–**6**). BAE exhibited consistently strong association with prediabetes, especially at 10th deciles (aOR=4.05, 95% CI: 3.02–5.42) (**Figure 6**). For IFG, the odds of predicted lean body mass exceeded that of the others (D10: aOR=3.34, 95% CI: 1.92–5.81) (**Figure 3**). Except predicted lean body mass, other 15 obesity indicators all presented significant associations with IGT, particularly at 9th and 10th deciles in predicted percent fat (aOR=5.28, 95% CI: 3.48–8.00; aOR=6.58, 95% CI: 4.33–10.00) (**Figure 4**). Correspondingly, only BMI, PI, CVAI, BAE, and predicted lean body mass made sense in detecting correlation with elevated HbA1c, in which predicted lean body mass presented the highest odds at 10th deciles (aOR=3.64, 95% CI: 1.92–6.91) (**Figures 5**, **6**).

## Discussion

DM, a silent killer, is a growing epidemic with disease burden increased globally (1, 41). Currently, DM and its complications constitute huge health burden in China (7). As prediabetes is a precursor before the diagnosis of DM, aiming people at high risk of prediabetes, identifying factors for the early screening of prediabetes, and implementing early prevention program could be cost effective and essential to break the vicious cycle of DM epidemic (8, 12, 13). Hence, knowledge on the correlated factors of corresponding high-risk groups classified by ADA diagnostic criteria of prediabetes was important for targeted and tailored prevention approaches. In our present study, we comprehensively identified and evaluated factors of demographic, behavioral, clinical, and biochemical characteristics, anthropometric characteristics, and anthropometric prediction equation that were correlated with prediabetes defined by different prediabetes criteria in the Chinese population. As far as we know, there are few studies that take full range of factors into consideration for prediabetes explicitly in a large Chinese population, particularly for the more extensive obesity indicators.

A meta-analysis that pooled five studies with three tests used (FPG, 2hPG, and HbA1c) showed that 8.7% prediabetes fulfilled all the three criteria of ADA (13). A study in Guangdong, China conducted in 2010 reported that the joint distribution of IGT, IFG, and elevated HbA1c was 5.2% (42). In addition, Gregory and colleagues revealed that the overlap in people with IGT, IFG, and elevated HbA1c levels was small (14). A similar finding of the overlap was also found in our present study. Comparable to the study of Gregory, with IGT showing the much lower proportion of prediabetes in their study (14), the elevated HbA1c took the lowest proportion in our study. Our present study suggested that the distribution of IFG, IGT, and elevated HbA1c of prediabetes in China was different from that in Europe, particular in gender, in which European women were mostly classified as prediabetes with the HbA1c criteria (13, 14), while prediabetic phenotype distributions under different diagnostic criteria stratified by gender were consistent with the total observations in our study. Therefore, it might be eligible to carry out certain types of prevention for these different prediabetic groups in China.

A considerable number of studies have found that the risk of prediabetes increased with the increase in age (16, 43), which was similar to our present study. The most likely pathophysiological reason could be that the human body became less sensitive to insulin and the β cells got altered insulin production with advancing age (44). Additionally, our study found that higher age groups were more likely to suffer from IGT and HbA1c, except IFG. These findings were partially consistent with the study of Gregory, in which the association between IFG and age was also detected in their study (14). It was reported that there were 17.1 million more men diagnosed with DM than women (16), and our study also presented that women had lower odds for total prediabetes when compared with men. Nevertheless, compared with men, a higher odd in women was detected for IGT. To date, no study has revealed the association between gender and IGT separately in the Chinese population. Income and smoking status in our study were found to have no statistical association with prediabetes in the multivariable analysis, which was consistent with a European study (14). Compared with previous studies, we also found that other demographic characteristics, such as educational level, drug history, family history, and regular exercise, presented similar associations with prediabetes (14, 16, 43, 45). It is suggested that alcohol was a risk factor for DM when consumed above a certain threshold value, and it reduced the risk of DM when below the threshold value (46). A European study found no statistical association between alcohol consumption and prediabetes, and only a decreased risk was detected between alcohol consumption and elevated HbA1c (14). Our study presented that a never drinking status was associated with a reduced risk of prediabetes. However, an increased risk was detected for elevated HbA1c phenotype when compared with people who ever drank alcohol. Nevertheless, Shinya and colleagues conducted a study to estimate the effect of alcohol consumption on the plasma glucose in non-diabetic men and found that as the level of alcohol consumption increased, the plasma glucose levels rose, but the HbA1c level was lower, which was partially verified by our findings (46). Although there was controversy on the topic of alcohol consumption, alcohol consumption negatively impacted diabetes self-care adherence (47). We found that hypertension was correlated with increased risk for prediabetes, which was consistent with previous studies (14, 45). For the aspect of dyslipidemia, a Spanish study reported the association of TC, LDL-c, and TG with IFG (48). A study of Mexican-Americans detected that HDL-c and TG were related to prediabetes, and no associations were found in TC and LDL-c (43). In our study, only TC, LDL-c, and TG presented correlations with prediabetes, and inconsistencies existed among certain prediabetic phenotypes.

Our results regarding the association between prediabetes and certain obesity indicators, which were well-studied recently, such as WC, WHtR, WHR, and BM, were consistent with previous findings where increases in WC, WHtR, WHR, and BMI were correlated with increased odds of prediabetes (45). However, we found that WC, WHtR, and WHR all failed to detect the association with the elevated HbA1c. Remarkably, obesity has been associated with an increased risk of T2DM, and the obese phenotypes were complex (36). It was suggested that the risk of obesity did not lie so much in the amount of fat accumulated but in its distribution (36). WC was a major clinical parameter for indirect evaluation of increased visceral fat, but it did not contribute to distinguish between subcutaneous and visceral fat mass (29). However, none of the anthropometric indices were considered a sufficiently accurate method for the estimation of adiposity and its distribution. To the best of our knowledge, it was the first study to take these anthropometric indices into account comprehensively, which estimated the associations in every certain prediabetic phenotypes in the Chinese population. In addition, it could contribute to the evaluation of the predictive capacity of these indicators to identify people with high risk of prediabetes. Although a recent study reported that a specific PI growth trajectory pattern during adolescence might be critical for diabetes prevention efforts (49), few studies examined the association between PI and prediabetes in adults. Conicity index was considered as a simple anthropometric index of body fat distribution to assess the central obesity with high accuracy (25). Mirelli and colleagues reported that the conicity index was an important tool in estimating the risk of DM in women (50). In our present study, we found that the conicity index was correlated with IFG and IGT, except elevated HbA1c group. RFM, as a new estimator of whole-body fat percentage, provided high predictability for dyslipidemias and metabolic syndrome (51). A cohort study suggested that RFM was confirmed to outperform BMI in predicting overall mortality but was not superior to WHR or WC (52). Our study presented that RFM showed stronger correlation than WC, WHtR, WHR, and BMI for prediabetes but failed to detected association with the elevated HbA1c. Previous studies suggested that significant positive correlations were observed between AVI, LAP, BRI, BAE, and DM (53–56), but few studies have focused on the Chinese population and prediabetes. BAE showed the strongest correlation than other indicators for total prediabetes in our study. A study for discriminating prediabetes/diabetes in the German population showed that VAI is a useful index for identifying prediabetes/diabetes in both men and women, as it is a valuable indictor of visceral adipose function (57), whereas no significant association between VAI and prediabetes was found in our study. The pronounced differences in body fat distribution existed among various ethnicities (58). It was worth mentioning that VAI seemed not suitable for the Chinese, as the Asian population is characterized by relatively higher body fat content at lower BMI values when compared with Caucasians (30). In contrast to VAI, CVAI was created for the Chinese population (30), and it was found to be correlated with prediabetes in our study. Furthermore, anthropometric prediction equation was applied and found that predicted fat mass demonstrated consistently stronger association with DM risk in the US people (33). However, the association between other indicators of anthropometric prediction equation and prediabetes remained absent. In our study, the odds of predicted lean body mass exceed other indicators for IFG and elevated HbA1c, and predicted percent fat presented the highest odds for IGT. Our results of obesity indicators provide new insights on the relationship between obesity and prediabetes, which in turn could help improve clinical and public health intervention for early prediabetes prevention.

There was no doubt that prevention approaches in Chinese individuals should differ from those advocated in other populations currently (4). The precise knowledge of prediabetes was not only helpful to distinguish people who might develop prediabetes from those who might not (15) but also contribute to self-identification and self-management. Thus, targeted and regular screening for people at high risk of prediabetes could be cost saving than universal screening (13, 14). Meanwhile, intervention *via* lifestyle changes of these people might be of importance to provide an excellent opportunity in the early prevention to reduce the prevalence and burden of DM. Moreover, the different criteria only showed limited overlap of prediabetes population in our study, which may indicate that people with different pathophysiologies were identified, as IGT and IFG have a different underlying pathophysiology (8). It is likely that the efficacy of different preventive treatments may differ for these subgroups. Thus, personalized interventions targeting these factors and measures were needed among Chinese adults. Obviously, the prediabetic population was far from being homogeneous, and phenotyping it into less heterogeneous groups might prove useful for long-term risk assessment, follow-up, and primary prevention (15). Several merits of our study deserve to be pointed. First, the population-based design, standardized data collection methods, and strict laboratory measurement with all the three tests used (FPG, 2hPG, and HbA1c) insured the reliability of the data, which permitted accurate estimates for the factors. Second, application of the GEE models provided an opportunity to power the overall study and prediabetes diagnostic criteria-specific analyses. Third, a broad set of obesity indicators were systematically evaluated in our study, incompletely characterized in previous studies, which were pragmatic and easy-to-measure characteristics as potential predictors for targeted screening and personalized intervention. However, some limitations existed. First, potential bias could not be avoided in our study, as information on behavioral characteristics was self-reported. Some unknown confounders cannot be avoided as with any observational study. Second, due to the lack of information on dietary factors and psychological stress, we could not evaluate them in our present study. Third, a majority of the participants in our study were rural inhabitants and of Han nationality. Consequently, speculation on the general population of China should be interpreted with caution.

Overall, 58.42% observations were prediabetes. The proportion of prediabetes with IFG, IGT, and elevated HbA1c was 9.66%. Despite demographic, behavioral, clinical, and biochemical characteristics, obesity indicators were also easily measured for target identification. Some correlated factors among IFG, IGT, and elevated HbA1c differed. Our findings could be used for targeted intervention to optimize prevention to mitigate the obviously increased prevalence of DM.

## Data availability statement

The datasets presented in this article are not readily available because of the requirements of this project. Requests to access the datasets should be directed to wangbeilxb@163.com.

## Ethics statement

This study was reviewed and approved by Ethics Review Committee of Jiangsu Provincial Centre for Disease Control and Privation and Zhongda Hospital, Southeast University (JSJK2017-B003-02). The patients/participants provided their written informed consent to participate in this study.

## Author contributions

All authors certified that they have participated in the conceptual design of this work, the analysis of the data, and the writing of the manuscript to take public responsibility for it. All authors reviewed the final version of the manuscript and approve it for publication. Conceptualization, BW. Methodology, XZ. Software, XZ. Validation, ZH, LC, and TXY. Formal analysis, XZ, HB, and RL. Investigation, JH, ML, and TY. Data curation, YOL and CS. Writing—original draft preparation, XZ. Writing—review and editing, BW and XZ. Visualization, ZY. Supervision, ZS. Project administration, HG, KS, and YL.

## Funding

This research has received funding from the National Key Research and Development Program of China (Grant No. 2016YFC1305700).

## Acknowledgments

We would like to express our sincere gratitude to the health workers and participants in this National Key Research and Development Program of China for their considerable efforts and collaboration.

## Conflict of interest

The authors declare that the research was conducted in the absence of any commercial or financial relationships that could be construed as a potential conflict of interest.

## Publisher’s note

All claims expressed in this article are solely those of the authors and do not necessarily represent those of their affiliated organizations, or those of the publisher, the editors and the reviewers. Any product that may be evaluated in this article, or claim that may be made by its manufacturer, is not guaranteed or endorsed by the publisher.

## Abbreviations

DM, diabetes mellitus; T2DM, type 2 diabetes mellitus; IFG, impaired fasting glucose; IGT, impaired glucose tolerance; FPG, fasting plasma glucose; 2hPG, 2-h postprandial glucose; HbA1c, glycosylated hemoglobin; TC, total cholesterol; LDL-c, low-density lipoprotein cholesterol; HDL-c, high-density lipoprotein cholesterol; TG, triglyceride; WC, waist circumference; WHtR, waist-to-height ratio; WHR, waist-to-hip ratio; BMI, body mass index; PI, ponderal index; RFM, relative fat mass; AVI, abdominal volume index; LAP, lipid accumulation product; VAI, visceral adiposity index; CVAI, Chinese visceral adiposity index; BRI, body roundness index; BAE, body adiposity estimator; ADA: American Diabetes Association; SDs, standard deviations; GEE, generalized estimating equation; ORs, odd ratios; CIs, confidence intervals.
