# Supplementary material for: Factors correlated with targeted prevention for prediabetes classified by impaired fasting glucose, impaired glucose tolerance, and elevated HbA1c: A population-based longitudinal study
Source: Front Endocrinol (Lausanne). 2022 Aug 22;13:965890. doi: 10.3389/fendo.2022.965890 (PMC9441664; doi:10.3389/fendo.2022.965890)
Supplement: Supplementary file 1 [file DataSheet_1.docx]

**Supplementary material 1**

**Factors correlated with targeted prevention for prediabetes classified by impaired fasting glucose, impaired glucose tolerance and elevated HbA1c: A population-based longitudinal study**


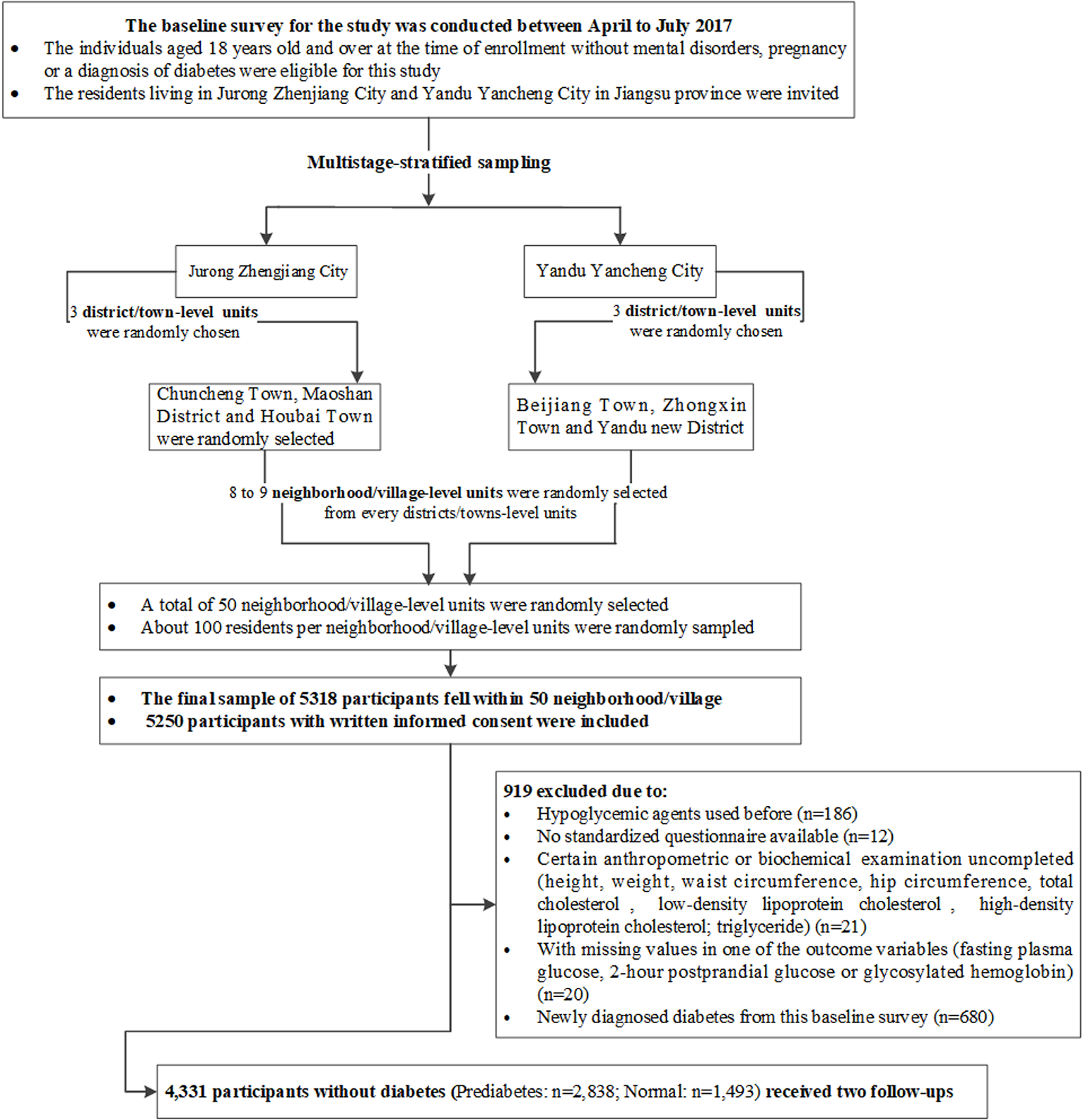


**Figure S1 Participant flowchart of baseline survey**


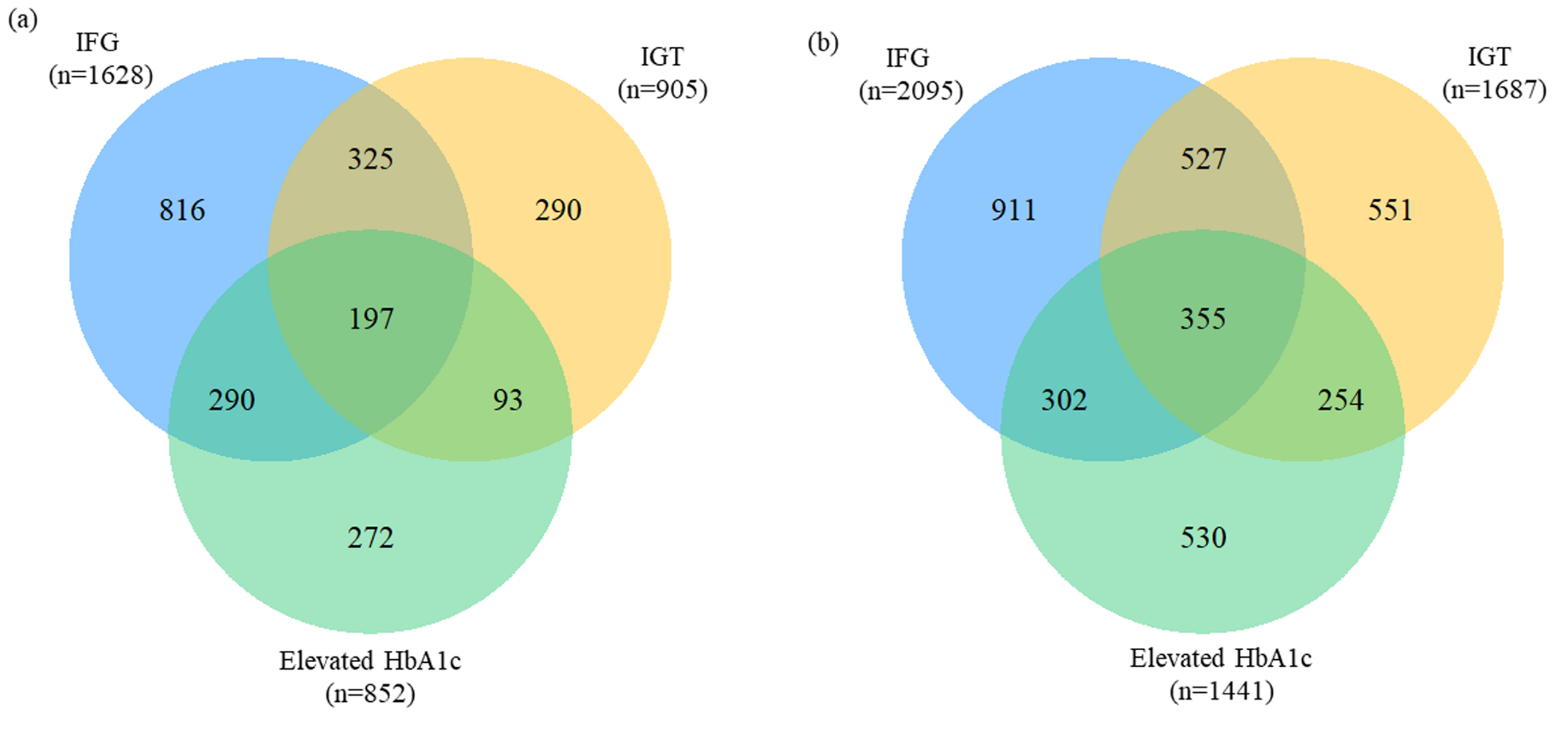


**Figure S2 Venn diagram for the overlap of prediabetes criteria stratified by gender**

1. Prediabetes of the male (n=2283); (b) Prediabetes of the female (n=3430)

| **Table S1 Estimations of the correlations among outcome measurements of every follow-up time** | | | | |
| --- | --- | --- | --- | --- |
| **Outcome measurements** | **Variables 1** | **Variables 2** | **r** | ***P* for Contingency Coefficients** |
| FPG level | FPG in 2017 | FPG in 2018 | 0.53 | <0.01 |
|  | FPG in 2017 | FPG in 2020 | 0.50 | <0.01 |
|  | FPG in 2018 | FPG in 2020 | 0.53 | <0.01 |
| 2hPG | 2hPG in 2017 | 2hPG in 2018 | 0.49 | <0.01 |
|  | 2hPG in 2017 | 2hPG in 2020 | 0.48 | <0.01 |
|  | 2hPG in 2018 | 2hPG in 2020 | 0.48 | <0.01 |
| HbA1c (%) | HbA1c in 2017 | HbA1c in 2018 | 0.59 | <0.01 |
|  | HbA1c in 2018 | HbA1c in 2020 | 0.59 | <0.01 |
|  | HbA1c in 2018 | HbA1c in 2020 | 0.62 | <0.01 |
| FPG: fasting plasma glucose; 2hPG: 2-hour postprandial glucose; HbA1c: glycosylated hemoglobin. | | | | |

| **Table S2 Baseline characteristics of the population without diabetes** | | | | | | | |
| --- | --- | --- | --- | --- | --- | --- | --- |
| **Variables** | **Criterion of prediabetes** | | | **Prediabetes ^a^ (n=2838)** | **Normal ^b^**  **(n=1493)** | **t/χ^2^** | ***P**** |
|  | **IFG**  **(n=2305)** | **IGT**  **(n=1140)** | **Elevated HbA1c (n=901)** |  |  |  |  |
| **Demographic characteristics** |  |  |  |  |  |  |  |
| **Age (y), n (%)** |  |  |  |  |  |  |  |
| (mean±SD) | 52.18±8.65 | 52.47±8.31 | 53.59±7.52 | 52.03±8.61 | 48.64±9.97 | 11.68 | <0.01 |
| <50 | 791 (34.32) | 379 (33.24) | 233 (25.86) | 990 (34.88) | 743 (49.77) | 98.05 | <0.01 |
| 50-59 | 939 (40.74) | 471 (41.32) | 428 (47.50) | 1162 (40.95) | 517 (34.63) |  |  |
| ≥60 | 575 (24.94) | 290 (25.44) | 240 (26.64) | 686 (24.17) | 233 (15.61) |  |  |
| **Gender, n (%)** |  |  |  |  |  |  |  |
| Male | 1005 (43.60) | 390 (34.21) | 344 (38.18) | 1179 (41.54) | 493 (33.02) | 29.98 | <0.01 |
| Female | 1300 (56.40) | 750 (65.79) | 557 (61.82) | 1659 (58.46) | 1000 (66.98) |  |  |
| **Education level, n (%)** |  |  |  |  |  |  |  |
| Junior high school or below | 1963 (85.16) | 998 (87.54) | 788 (87.46) | 2430 (85.62) | 1212 (81.18) | 14.45 | <0.01 |
| Senior high school or above | 342 (14.84) | 142 (12.46) | 113 (12.54) | 408 (14.38) | 281 (18.82) |  |  |
| **Equivalent household income, n (%)** |  |  |  |  |  |  |  |
| Low | 767 (33.27) | 388 (34.03) | 312 (34.63) | 943 (33.23) | 452 (30.27) | 11.62 | <0.01 |
| Moderate | 824 (35.75) | 418 (36.67) | 324 (35.96) | 1011 (35.62) | 500 (33.49) |  |  |
| High | 714 (30.98) | 334 (29.30) | 265 (29.41) | 884 (31.15) | 541 (36.24) |  |  |
| **Drug history, n (%)** |  |  |  |  |  |  |  |
| No | 1630 (70.72) | 761 (66.75) | 598 (66.37) | 2026 (71.39) | 1240 (83.05) | 71.80 | <0.01 |
| Yes | 675 (29.28) | 379 (33.25) | 303 (33.63) | 812 (28.61) | 253 (16.95) |  |  |
| **Family history of diabetes, n (%)** |  |  |  |  |  |  |  |
| No | 1817 (78.83) | 857 (75.17) | 704 (78.14) | 2221 (78.26) | 1186 (79.44) | 1.51 | 0.47 |
| Yes | 400 (17.35) | 224 (19.65) | 165 (18.31) | 497 (17.51) | 240 (16.07) |  |  |
| Unclear | 88 (3.82) | 59 (5.18) | 32 (3.55) | 120 (4.23) | 67 (4.49) |  |  |
| **Behavioral characteristics** |  |  |  |  |  |  |  |
| **Smoking status, n (%)** |  |  |  |  |  |  |  |
| Non-smoker | 1691 (73.36) | 887 (77.81) | 665 (73.81) | 2112 (74.42) | 1191 (79.77) | 24.68 | <0.01 |
| Current smoker | 500 (21.69) | 204 (17.89) | 197 (21.86) | 600 (21.14) | 273 (18.29) |  |  |
| Ex-smoker | 114 (4.95) | 49 (4.30) | 39 (4.33) | 126 (4.44) | 29 (1.94) |  |  |
| **Drinking status, n (%)** |  |  |  |  |  |  |  |
| Ever | 688 (29.85) | 291 (25.53) | 242 (26.86) | 815 (28.72) | 295 (19.76) | 41.19 | <0.01 |
| Never | 1617 (70.15) | 849 (74.47) | 659 (73.14) | 2023 (71.28) | 1198 (80.24) |  |  |
| **Regular exercise, n (%)** |  |  |  |  |  |  |  |
| Yes | 606 (26.29) | 320 (28.07) | 243 (26.97) | 746 (26.29) | 408 (27.33) | 0.54 | 0.46 |
| No | 1699 (73.71) | 820 (71.93) | 658 (73.03) | 2092 (73.71) | 1085 (72.67) |  |  |
| **Clinical and biochemical characteristics** |  |  |  |  |  |  |  |
| **Hypertension, n (%) ^c^** |  |  |  |  |  |  |  |
| No | 1399 (60.77) | 638 (56.01) | 516 (57.46) | 1733 (61.17) | 1127 (75.79) | 93.14 | <0.01 |
| Yes | 903 (39.23) | 501 (43.99) | 382 (42.54) | 1100 (38.83) | 360 (24.21) |  |  |
| **TC (mmol/L)** (mean±SD) | 4.87±0.91 | 4.97±0.93 | 5.05±1.01 | 4.88±0.95 | 4.65±0.87 | 8.03 | <0.01 |
| **LDL-C (mmol/L)** (mean±SD) | 2.67±0.69 | 2.73±0.68 | 2.78±0.72 | 2.67±0.69 | 2.53±0.66 | 6.57 | <0.01 |
| **HDL-C (mmol/L)** (mean±SD) | 1.52±0.37 | 1.49±0.38 | 1.51±0.37 | 1.52±0.37 | 1.53±0.35 | 0.94 | 0.34 |
| **TG (mmol/L)** (median, IQR) | 1.36 (0.95-2.11) | 1.52 (1.02-2.22) | 1.37 (0.95-2.03) | 1.36 (0.95-2.07) | 1.16 (0.85-1.68) | — | <0.01^Ϯ^ |
| **Anthropometric characteristics** |  |  |  |  |  |  |  |
| **WC (cm)** (mean±SD) | 85.57±9.55 | 86.28±9.93 | 84.79±9.55 | 85.48±9.53 | 83.27±9.24 | 7.33 | <0.01 |
| **WHtR** (median, IQR) | 0.53 (0.49-0.57) | 0.54 (0.51-0.58) | 0.49 (0.53-0.57) | 0.53 (0.49-0.57) | 0.52 (0.48-0.55) | — | <0.01^Ϯ^ |
| **WHR** (median, IQR) | 0.90 (0.86-0.94) | 0.90 (0.86-0.94) | 0.89 (0.86-0.93) | 0.90 (0.86-0.93) | 0.88 (0.84-0.91) | — | <0.01^Ϯ^ |
| **BMI** (kg/m^2^) (mean±SD) | 25.70±3.50 | 26.36±3.69 | 25.94±3.45 | 25.70±3.49 | 24.60±3.36 | 9.93 | <0.01 |
| **PI** (kg/m^3^) (median, IQR) | 15.81 (14.43-17.41) | 16.42 (14.92-18.05) | 16.15 (14.68-17.65) | 15.85 (14.45-17.45) | 15.17 (13.83-16.68) | — | <0.01^Ϯ^ |
| **CI** (m^3/2^·kg^1/2^) (mean±SD) | 50.65±8.78 | 51.47±9.12 | 50.26±8.47 | 50.55±8.72 | 48.18±8.47 | 8.60 | <0.01 |
| **RFM** (median, IQR) | 33.33 (26.73-39.26) | 36.27 (28.54-40.43) | 35.15 (27.04-39.45) | 34.13 (26.89-39.33) | 34.67 (27.38-38.72) | — | 0.77^Ϯ^ |
| **AVI** (L) (median, IQR) | 14.65 (12.66-16.95) | 14.84 (12.85-17.05) | 14.51 (12.31-16.68) | 14.61 (12.64-16.94) | 13.90 (12.02-16.00) | — | <0.01^Ϯ^ |
| **LAP** (cm·mmol/L) (median, IQR) | 33.60 (19.26-56.00) | 39.29 (22.97-62.58) | 32.70 (18.69-55.16) | 33.57 (19.35-55.89) | 25.80 (15.96-43.92) | — | <0.01^Ϯ^ |
| **VAI** (median, IQR) | 1.45 (0.91-2.41) | 1.74 (1.05-2.73) | 1.44 (0.91-2.44) | 1.45 (0.91-2.41) | 1.27 (0.85-1.99) | — | <0.01^Ϯ^ |
| **CVAI** (mean±SD) | 95.47±37.67 | 101.13±37.38 | 96.25±37.08 | 95.18±37.43 | 80.69±37.66 | 12.08 | <0.01 |
| **BRI** (median, IQR) | 4.01 (3.24-4.74) | 4.16 (3.46-4.97) | 3.99 (3.24-4.76) | 4.01 (3.25-4.74) | 3.71 (3.05-4.45) | — | <0.01^Ϯ^ |
| **BAE** (median, IQR) | 33.04 (26.58-38.57) | 35.67 (29.26-40.04) | 35.05 (28.03-39.36) | 33.61 (26.80-38.76) | 33.07 (26.47-37.13) | — | <0.01^Ϯ^ |
| **Anthropometric prediction equation ^d^** |  |  |  |  |  |  |  |
| **Lean body mass** (kg) (median, IQR) | 40.53 (35.66-50.11) | 39.13 (35.17-48.12) | 39.69 (35.42-49.20) | 40.11 (35.50-49.50) | 37.82 (34.46-46.58) | — | <0.01^Ϯ^ |
| **Fat mass** (kg) (median, IQR) | 22.29 (18.11-26.46) | 23.73 (19.61-27.79) | 22.96 (18.61-26.90) | 22.46 (18.28-26.50) | 21.40 (17.65-25.15) | — | <0.01^Ϯ^ |
| **Percent fat** (%) (median, IQR) | 35.50 (26.83-39.57) | 37.56 (28.97-40.61) | 37.10 (27.08-40.05) | 36.08 (27.00-39.64) | 35.92 (27.97-38.69) | — | 0.66^Ϯ^ |
| Data was presented as mean±SD (standard deviation) or median (IQR) for continuous variables when appropriate; number (percentage) for categorical variables; | | | | | | | |
| *χ^2^ test, t test or Wilcoxon test was used to examine the difference between prediabetes and the normal group;  ^Ϯ^ Wilcoxon test was used because of heterogeneity of variance; | | | | | | | |
| a. Prediabetes: IFG or IGT or elevated HbA1c; | | | | | | | |
| b. Normal: without IFG, IGT or elevated HbA1c; | | | | | | | |
| c. 5 missing value in this characteristic; | | | | | | | |
| d. Lean body mass, fat mass and percent fat were derived from a validated anthropometric prediction equation; | | | | | | | |
| IFG: impaired fasting glucose; IGT: impaired glucose tolerance; HbA1c: glycosylated hemoglobin; TC: total cholesterol; LDL-C: low-density lipoprotein cholesterol; HDL-C: high-density lipoprotein cholesterol; TG: triglyceride; IQR, inter-quartile range; WC: waist circumference; WHtR: waist-to-height ratio; WHR: waist-to-hip ratio; BMI: body mass index; PI: ponderal index; CI: conicity index; RFM: relative fat mass; AVI: abdominal volume index; LAP: lipid accumulation product; VAI: visceral adiposity index; CVAI: Chinese visceral adiposity index; BRI: body roundness index; BAE: body adiposity estimator. | | | | | | | |

| **Table S3 Deciles of obesity indicators** | | | | | | | | | | |  |
| --- | --- | --- | --- | --- | --- | --- | --- | --- | --- | --- | --- |
| **Obesity indicators** | **D1** | **D2** | **D3** | **D4** | **D5** | **D6** | **D7** | **D8** | **D9** | **D10** | |
| **WC** (cm) | <72.00 | 72.00-75.59 | 76.00-78.99 | 79.00-81.89 | 81.90-83.99 | 84.00-86.49 | 86.50-91.99 | 92.00-95.99 | 96.00-166.49 | ≥166.50 |  |
| **WHtR** | <0.45 | 0.45-0.47 | 0.48-0.48 | 0.49-0.50 | 0.51-0.52 | 0.53 | 0.54 | 0.55-0.56 | 0.57-0.59 | ≥0.60 |  |
| **WHR** | <0.81 | 0.81-0.83 | 0.84-0.85 | 0.86 | 0.87-0.88 | 0.89 | 0.90-0.91 | 0.92-0.93 | 0.94-0.95 | ≥0.96 |  |
| **BMI** (kg/m^2^) | <20.91 | 20.91-22.18 | 22.19-23.15 | 23.16-24.06 | 24.07-24.88 | 24.89-25.70 | 25.71-26.63 | 26.64-27.71 | 27.72-29.46 | ≥29.47 |  |
| **PI** (kg/m^3^) | <12.92 | 12.92-13.77 | 13.78-14.42 | 14.43-14.95 | 14.96-15.51 | 15.52-16.09 | 16.10-16.69 | 16.70-17.47 | 17.48-18.61 | ≥18.62 |  |
| **CI** (m^3/2^·kg^1/2^) | <38.54 | 38.54-41.72 | 41.73-44.26 | 44.27-46.45 | 46.46-48.54 | 48.55-50.77 | 50.78-53.22 | 53.23-56.11 | 56.12-60.29 | ≥60.30 |  |
| **RFM** | <22.99 | 22.99-25.99 | 26.00-28.16 | 28.17-30.92 | 30.93-34.17 | 34.18-36.48 | 36.49-38.19 | 38.20-39.87 | 39.88-41.92 | <41.93 |  |
| **AVI** (L) | <10.55 | 10.55-11.70 | 11.71-12.64 | 12.65-13.45 | 13.46-14.19 | 14.20-15.01 | 15.02-15.93 | 15.94-16.95 | 16.96-18.49 | ≥18.50 |  |
| **LAP** (cm·mmol/L) | <10.08 | 10.08-15.44 | 15.45-20.15 | 20.16-25.13 | 25.14-30.74 | 30.75-37.79 | 37.80-46.38 | 46.39-58.91 | 58.92-83.75 | ≥83.76 |  |
| **VAI** | <0.66 | 0.66-0.85 | 0.86-1.04 | 1.05-1.24 | 1.25-1.46 | 1.47-1.74 | 1.75-2.11 | 2.12-2.64 | 2.65-3.76 | ≥3.77 |  |
| **CVAI** | <43.50 | 43.50-59.87 | 59.88-72.01 | 72.02-82.35 | 82.36-92.11 | 92.12-101.44 | 101.45-110.57 | 110.58-121.72 | 121.73-137.48 | ≥137.49 |  |
| **BRI** | <2.49 | 2.49-2.92 | 2.93-3.24 | 3.25-3.54 | 3.55-3.84 | 3.85-4.11 | 4.12-4.43 | 4.44-4.81 | 4.82-5.40 | ≥5.41 |  |
| **BAE** | <22.63 | 22.63-25.62 | 25.63-28.23 | 28.24-30.95 | 30.96-33.45 | 33.46-35.39 | 35.40-37.18 | 37.19-39.02 | 39.03-41.34 | ≥41.35 |  |
| **Lean body mass** (kg)**^*^** | <32.21 | 32.21-33.99 | 34.00-35.45 | 35.46-36.93 | 36.94-38.76 | 38.77-41.57 | 41.58-45.72 | 45.73-49.82 | 49.83-53.83 | ≥53.84 |  |
| **Fat mass** (kg)**^*^** | <14.08 | 14.08-16.79 | 16.80-18.66 | 18.67-20.27 | 20.28-21.75 | 21.76-23.18 | 23.19-24.79 | 24.80-26.77 | 26.78-29.76 | ≥29.77 |  |
| **Percent fat** (%)**^*^** | <23.29 | 23.29-26.05 | 26.06-28.82 | 28.83-33.55 | 33.56-35.99 | 36.00-37.39 | 37.40-38.66 | 38.67-39.95 | 39.96-41.70 | ≥41.71 |  |
| D: decile; WC: waist circumference; WHtR: waist-to-height ratio; WHR: waist-to-hip ratio; BMI: body mass index; PI: ponderal index; CI: conicity index; RFM: relative fat mass; AVI: abdominal volume index; LAP: lipid accumulation product; VAI: visceral adiposity index; CVAI: Chinese visceral adiposity index; BRI: body roundness index; BAE: body adiposity estimator; | | | | | | | | | | |  |
| *Lean body mass, fat mass and percent fat were derived from a validated anthropometric prediction equation. | | | | | | | | | | |  |
