# Supplementary material for: Factors correlated with targeted prevention for prediabetes classified by impaired fasting glucose, impaired glucose tolerance, and elevated HbA1c: A population-based longitudinal study
Source: Front Endocrinol (Lausanne). 2022 Aug 22;13:965890. doi: 10.3389/fendo.2022.965890 (PMC9441664; doi:10.3389/fendo.2022.965890)
Supplement: Supplementary file 2 [file DataSheet_2.docx]

**Supplementary material 2**

**Factors correlated with targeted prevention for prediabetes** **classified by impaired fasting glucose, impaired glucose tolerance and elevated HbA1c: A population-based longitudinal study**

**Equations of obesity indicators**

1. **Waist-to-height ratio, WHtR**(1)

$$\text{WHtR=}\frac{\text{WC}}{height}$$

1. **Waist-to-hip ratio, WHR**(2)

$$\text{WHR=}\frac{\text{WC}}{\text{Hip}}$$

1. **Body mass index, BMI**(3)

$BMI=\frac{weight}{height^{2}}$ ($\text{kg/m}^{2}$)

1. **Ponderal index, PI**(4)

**$\text{PI=}\frac{\text{weight}}{\text{height}^{3}}$** ($\text{kg/m}^{3}$)

1. **Conicity index, CI**(5)

$CI=\frac{WC}{0.109\sqrt{\frac{Weight}{Height}}}$ ()

1. **Relative fat mass, RFM**(6)

$$\text{RFM=64-(20}\times\text{(height/waist))+(12}\times\text{sex)}$$

(Male=0; Female=1)

1. **Abdominal volume index, AVI**(7)

$AVI=\frac{[2\times waist^{2}+0.7\times(waist-hip)^{2}]}{1000}$ (L)

1. **Liquid accumulation product, LAP**(8)

$\text{LAP}_{\text{男}}\text{= [WC - 65] }\times\text{TG}$ ()

$\text{LAP}_{\text{女}}\text{= [WC - 58] }\times\text{TG}$ ()

1. **Visceral adiposity index, VAI**(9)

Males:$\text{VAI=(}\frac{\text{WC}}{39.68\text{+(1.88 }\times\text{ BMI)}}\text{) }\times\text{ (}\frac{\text{TG}}{1.03}\text{) }\times\text{ (}\frac{1.31}{\text{HDL}}\text{)}$

Females:$\text{VAI=(}\frac{\text{WC}}{36.58\text{+(1.89 }\times\text{ BMI)}}\text{) }\times\text{ (}\frac{\text{TG}}{0.81}\text{) }\times\text{ (}\frac{1.52}{\text{HDL}}\text{)}$

1. **Chinese visceral adiposity index, CVAI**(10)

Male:$\text{CVAI=-}267.93\text{+}0.68*\text{age+}0.03*\text{BMI+}4.00*\text{WC+22.00*Log}_{10}\text{TG-}16.32\text{*HDL-C}$ Females:$\text{CVAI=-187.32+1.71}*\text{age+4.23}*\text{BMI+1.12}*\text{WC+39.76*Log}_{10}\text{TG-}11.66\text{*HDL-C}$

1. **Body roundness index, BRI**(11)

$$BRI=364.2-365.5\times\sqrt{1-\left(\frac{(WC/(2\pi))^{2}}{(0.5*height)^{2}}\right)}$$

1. **Body adiposity estimator, BAE**(12)

$$\begin{matrix} \text{BAE=-44.988+(0.503}\times\text{age)+(10.689}\times\text{sex)+(3.172}\times\text{BMI)-(0.026}\times\text{BMI}^{2}\text{)+} \\ \text{(0.81}\times\text{BMI}\times\text{sex)-(0.02}\times\text{BMI}\times\text{age)-(0.005}\times\text{BMI}^{2}\times\text{sex)+(0.00021}\times\text{BMI}^{2}\times\text{age)} \end{matrix}$$

(Male=0; Female=1)

1. **Anthropometric prediction equation**(13)

Lean body mass (kg)

Males:

$$\begin{matrix} \text{LBM=19.363+0.001}\times\text{age+0.064}\times\text{height+0.756}\times\text{weight-0.366}\times\text{waist} \\ \text{-0.066}\times\text{Mexican+0.231}\times\text{Hispanic+0.432}\times\text{Blanck-1.007}\times\text{other ethnicity} \end{matrix}$$

Females:

$$\begin{matrix} \text{LBM=-10.683-0.039}\times\text{age+0.186}\times\text{height+0.383}\times\text{weight-0.043}\times\text{waist} \\ \text{-0.359}\times\text{Mexican-0.059}\times\text{Hispanic+1.085}\times\text{Blanck-0.34}\times\text{other ethnicity} \end{matrix}$$

Fat mass (kg)

Males:

$$\begin{matrix} \text{LBM=-18.592-0.009}\times\text{age-0.080}\times\text{height+0.226}\times\text{weight+0.387}\times\text{waist} \\ \text{+0.080}\times\text{Mexican-0.188}\times\text{Hispanic-0.483}\times\text{Blanck+1.050}\times\text{other ethnicity} \end{matrix}$$

Females:

$$\begin{matrix} \text{LBM=11.817+0.041}\times\text{age-0.199}\times\text{height+0.610}\times\text{weight+0.044}\times\text{waist} \\ \text{+0.388}\times\text{Mexican+0.073}\times\text{Hispanic-1.187}\times\text{Blanck+0.325}\times\text{other ethnicity} \end{matrix}$$

Percent fat (%)

Males:

$$\begin{matrix} \text{LBM=0.02+0.00}\times\text{age-0.07}\times\text{height-0.08}\times\text{weight+0.48}\times\text{waist} \\ \text{+0.32}\times\text{Mexican+0.02}\times\text{Hispanic-0.65}\times\text{Blanck+1.12}\times\text{other ethnicity} \end{matrix}$$

Females:

$$\begin{matrix} \text{LBM=50.46+0.07}\times\text{age-0.26}\times\text{height+0.27}\times\text{weight+0.10}\times\text{waist} \\ \text{+0.89}\times\text{Mexican+0.49}\times\text{Hispanic-1.57}\times\text{Blanck+0.43}\times\text{other ethnicity} \end{matrix}$$

**References**

1. L. M. Browning, S. D. Hsieh and M. Ashwell: A systematic review of waist-to-height ratio as a screening tool for the prediction of cardiovascular disease and diabetes: 0.5 could be a suitable global boundary value. *Nutr Res Rev*, 23(2), 247-69 (2010) doi:10.1017/S0954422410000144

2. M. Dalton, A. J. Cameron, P. Z. Zimmet, J. E. Shaw, D. Jolley, D. W. Dunstan, T. A. Welborn and C. AusDiab Steering: Waist circumference, waist-hip ratio and body mass index and their correlation with cardiovascular disease risk factors in Australian adults. *J Intern Med*, 254(6), 555-63 (2003) doi:10.1111/j.1365-2796.2003.01229.x

3. B. F. Zhou: Predictive values of body mass index and waist circumference for risk factors of certain related diseases in Chinese adults--study on optimal cut-off points of body mass index and waist circumference in Chinese adults. *Biomed Environ Sci*, 15(1), 83-96 (2002)

4. V. Florey Cdu: The use and interpretation of ponderal index and other weight-height ratios in epidemiological studies. *J Chronic Dis*, 23(2), 93-103 (1970) doi:10.1016/0021-9681(70)90068-8

5. R. Valdez: A simple model-based index of abdominal adiposity. *J Clin Epidemiol*, 44(9), 955-6 (1991) doi:10.1016/0895-4356(91)90059-i

6. O. O. Woolcott and R. N. Bergman: Relative fat mass (RFM) as a new estimator of whole-body fat percentage horizontal line A cross-sectional study in American adult individuals. *Sci Rep*, 8(1), 10980 (2018) doi:10.1038/s41598-018-29362-1

7. F. Guerrero-Romero and M. Rodriguez-Moran: Abdominal volume index. An anthropometry-based index for estimation of obesity is strongly related to impaired glucose tolerance and type 2 diabetes mellitus. *Arch Med Res*, 34(5), 428-32 (2003) doi:10.1016/S0188-4409(03)00073-0

8. H. S. Kahn: The "lipid accumulation product" performs better than the body mass index for recognizing cardiovascular risk: a population-based comparison. *BMC Cardiovasc Disord*, 5, 26 (2005) doi:10.1186/1471-2261-5-26

9. M. C. Amato, C. Giordano, M. Galia, A. Criscimanna, S. Vitabile, M. Midiri, A. Galluzzo and G. AlkaMeSy Study: Visceral Adiposity Index: a reliable indicator of visceral fat function associated with cardiometabolic risk. *Diabetes Care*, 33(4), 920-2 (2010) doi:10.2337/dc09-1825

10. M. F. Xia, Y. Chen, H. D. Lin, H. Ma, X. M. Li, Q. Aleteng, Q. Li, D. Wang, Y. Hu, B. S. Pan, X. J. Li, X. Y. Li and X. Gao: A indicator of visceral adipose dysfunction to evaluate metabolic health in adult Chinese. *Sci Rep*, 6, 38214 (2016) doi:10.1038/srep38214

11. D. M. Thomas, C. Bredlau, A. Bosy-Westphal, M. Mueller, W. Shen, D. Gallagher, Y. Maeda, A. McDougall, C. M. Peterson, E. Ravussin and S. B. Heymsfield: Relationships between body roundness with body fat and visceral adipose tissue emerging from a new geometrical model. *Obesity (Silver Spring)*, 21(11), 2264-71 (2013) doi:10.1002/oby.20408

12. J. Gomez-Ambrosi, C. Silva, V. Catalan, A. Rodriguez, J. C. Galofre, J. Escalada, V. Valenti, F. Rotellar, S. Romero, B. Ramirez, J. Salvador and G. Fruhbeck: Clinical usefulness of a new equation for estimating body fat. *Diabetes Care*, 35(2), 383-8 (2012) doi:10.2337/dc11-1334

13. D. H. Lee, N. Keum, F. B. Hu, E. J. Orav, E. B. Rimm, Q. Sun, W. C. Willett and E. L. Giovannucci: Development and validation of anthropometric prediction equations for lean body mass, fat mass and percent fat in adults using the National Health and Nutrition Examination Survey (NHANES) 1999-2006. *Br J Nutr*, 118(10), 858-866 (2017) doi:10.1017/S0007114517002665
