# Supplementary material for: Factors correlated with targeted prevention for prediabetes classified by impaired fasting glucose, impaired glucose tolerance, and elevated HbA1c: A population-based longitudinal study
Source: Front Endocrinol (Lausanne). 2022 Aug 22;13:965890. doi: 10.3389/fendo.2022.965890 (PMC9441664; doi:10.3389/fendo.2022.965890)
Supplement: Supplementary file 3 [file DataSheet_3.docx]

**Supplementary material 3**

**Factors correlated with targeted prevention for prediabetes classified by impaired fasting glucose, impaired glucose tolerance and elevated HbA1c: A population-based longitudinal study**

| **Table S1 Factors correlated with prediabetes** | | | | | | | | | | |  |  |  |
| --- | --- | --- | --- | --- | --- | --- | --- | --- | --- | --- | --- | --- | --- |
| **Variables** | **Criterions of prediabetes** | | | | | | | | | |  | **Prediabetes ^a^** | |
|  | **IFG** | |  | **IGT** | | |  | | **Elevated HbA1c** | |  |  |  |
|  | **OR (95% CI)** | **aOR (95% CI)^*^** |  | **OR (95% CI)** | | **aOR (95% CI)^*^** |  | **OR (95% CI)** | | **aOR (95% CI)^*^** |  | **OR (95% CI)** | **aOR (95% CI)^*^** |
| **Demographic characteristics a** | |  |  |  | |  |  | |  |  |  |  |  |
| **Age (y)** |  |  |  |  |  | |  | |  |  |  |  |  |
| <50 | Reference | Reference |  | Reference | | Reference |  | | Reference | Reference |  | Reference | Reference |
| 50-59 | 1.14 (1.02-1.26) | 1.04 (0.93-1.16) |  | 1.31 (1.16-1.48) | | 1.18 (1.04-1.34) |  | | 2.28 (1.99-2.61) | 2.15 (1.87-2.48) |  | 1.46 (1.32-1.62) | 1.34 (1.20-1.49) |
| ≥60 | 1.25 (1.10-1.41) | 1.09 (0.95-1.25) |  | 1.79 (1.56-2.05) | | 1.74 (1.50-2.01) |  | | 2.91 (2.50-3.38) | 2.85 (2.42-3.35) |  | 1.92 (1.69-2.18) | 1.74 (1.52-2.00) |
| *P* for trend |  | 0.22 |  |  | | <0.01 |  | |  | <0.01 |  |  | <0.01 |
| **Gender** |  |  |  |  | |  |  | |  |  |  |  |  |
| Male | Reference | Reference |  | Reference | | Reference |  | | Reference | Reference |  | Reference | Reference |
| Female | 0.65 (0.59-0.71) | 0.70 (0.61-0.81) |  | 1.17 (1.05-1.31) | | 1.41 (1.20-1.65) |  | | 1.00 (0.90-1.13) | 1.02 (0.87-1.20) |  | 0.76 (0.69-0.84) | 0.83 (0.72-0.96) |
| *P* for trend |  | <0.01 |  |  | | <0.01 |  | |  | 0.80 |  |  | 0.01 |
| **Education level** |  |  |  |  | |  |  | |  |  |  |  |  |
| Junior high school or below | Reference | Reference |  | Reference | | Reference |  | | Reference | Reference |  | Reference | Reference |
| Senior high school or above | 0.92 (0.81-1.06) | 0.84 (0.72-0.97) |  | 0.80 (0.69-0.94) | | 0.92 (0.78-1.09) |  | | 0.77 (0.65-0.91) | 0.87 (0.73-1.03) |  | 0.79 (0.69-0.91) | 0.80 (0.69-0.92) |
| *P* for trend |  | 0.02 |  |  | | 0.33 |  | |  | 0.11 |  |  | <0.01 |
| **Equivalent household income** |  |  |  |  | |  |  | |  |  |  |  |  |
| Low | Reference | Reference |  | Reference | | Reference |  | | Reference | Reference |  | Reference | Reference |
| Moderate | 0.91 (0.82-1.01) | 0.90 (0.81-1.00) |  | 1.01 (0.91-1.13) | | 1.07 (0.95-1.19) |  | | 1.01 (0.90-1.14) | 1.06 (0.94-1.20) |  | 0.96 (0.87-1.06) | 0.98 (0.88-1.08) |
| High | 0.89 (0.80-0.98) | 0.88(0.78-0.99) |  | 0.83 (0.74-0.94) | | 0.97 (0.85-1.10) |  | | 0.81 (0.71-0.93) | 1.06 (0.92-1.21) |  | 0.82 (0.73-0.91) | 0.92 (0.82-1.03) |
| *P* for trend |  | 0.03 |  |  | | 0.66 |  | |  | 0.41 |  |  | 0.14 |
| **Drug history** |  |  |  |  | |  |  | |  |  |  |  |  |
| No | Reference | Reference |  | Reference | | Reference |  | | Reference | Reference |  | Reference | Reference |
| Yes | 1.42 (1.28-1.57) | 1.07 (0.93-1.23) |  | 1.64 (1.47-1.83) | | 1.09 (0.94-1.27) |  | | 1.64 (1.46-1.83) | 1.10 (0.95-1.29) |  | 1.65 (1.48-1.83) | 1.06 (0.92-1.23) |
| *P* for trend |  | 0.35 |  |  | | 0.25 |  | |  | 0.21 |  |  | 0.42 |
| **Family history of diabetes** |  |  |  |  | |  |  | |  |  |  |  |  |
| No | Reference | Reference |  | Reference | | Reference |  | | Reference | Reference |  | Reference | Reference |
| Yes | 1.08 (0.95-1.23) | 1.09 (0.95-1.24) |  | 1.24 (1.08-1.42) | | 1.32 (1.15-1.53) |  | | 1.19 (1.03-1.37) | 1.36 (1.17-1.58) |  | 1.14 (0.99-1.30) | 1.22 (1.07-1.40) |
| Unclear | 0.86 (0.68-1.10) | 0.84 (0.66-1.08) |  | 1.37 (1.06-1.76) | | 1.52 (1.17-1.97) |  | | 0.77 (0.58-1.02) | 0.84 (0.63-1.12) |  | 1.01 (0.79-1.29) | 1.05 (0.82-1.34) |
| *P* for trend |  | 0.87 |  |  | | <0.01 |  | |  | 0.06 |  |  | 0.03 |
| **Behavioral characteristics ^b^** |  |  |  |  | |  |  | |  |  |  |  |  |
| **Smoking status** |  |  |  |  | |  |  | |  |  |  |  |  |
| Non-smoker | Reference | Reference |  | Reference | | Reference |  | | Reference | Reference |  | Reference | Reference |
| Current smoker | 1.27 (1.13-1.43) | 0.87 (0.75-1.01) |  | 0.89 (0.78-1.02) | | 0.98 (0.83-1.17) |  | | 1.05 (0.92-1.20) | 1.11 (0.93-1.32) |  | 1.16 (1.03-1.31) | 0.89 (0.76-1.05) |
| Ex-smoker | 1.88 (1.50-2.36) | 1.25 (0.97-1.60) |  | 1.26 (1.00-1.60) | | 1.30 (0.99-1.70) |  | | 1.21 (0.93-1.57) | 1.21 (0.91-1.61) |  | 1.69 (1.33-2.16) | 1.28 (0.98-1.68) |
| *P* for trend |  | 0.77 |  |  | | 0.21 |  | |  | 0.13 |  |  | 0.57 |
| **Drinking status** |  |  |  |  | |  |  | |  |  |  |  |  |
| Ever | Reference | Reference |  | Reference | | Reference |  | | Reference | Reference |  | Reference | Reference |
| Never | 0.62 (0.56-0.69) | 0.72 (0.64-0.82) |  | 0.95 (0.85-1.07) | | 0.90 (0.79-1.03) |  | | 1.09 (0.96-1.23) | 1.19 (1.03-1.38) |  | 0.73 (0.66-0.81) | 0.82 (0.72-0.93) |
| *P* for trend |  | <0.01 |  |  | | 0.15 |  | |  | 0.02 |  |  | <0.01 |
| **Regular exercise** |  |  |  |  | |  |  | |  |  |  |  |  |
| Yes | Reference | Reference |  | Reference | | Reference |  | | Reference | Reference |  | Reference | Reference |
| No | 1.30 (1.21-1.41) | 1.34 (1.23-1.45) |  | 1.00 (0.92-1.09) | | 1.03 (0.94-1.12) |  | | 1.44 (1.31-1.57) | 1.47 (1.34-1.62) |  | 1.39 (1.29-1.50) | 1.43 (1.32-1.55) |
| *P* for trend |  | <0.01 |  |  | | 0.57 |  | |  | <0.01 |  |  | <0.01 |
| **Clinical and biochemical characteristics ^b^** |  |  |  |  | |  |  | |  |  |  |  |  |
| **Hypertension** |  |  |  |  | |  |  | |  |  |  |  |  |
| No | Reference | Reference |  | Reference | | Reference |  | | Reference | Reference |  | Reference | Reference |
| Yes | 1.49 (1.36-1.63) | 1.17 (1.02-1.34) |  | 1.69 (1.53-1.87) | | 1.32 (1.15-1.51) |  | | 1.62 (1.46-1.80) | 1.25 (1.08-1.45) |  | 1.76 (1.60-1.93) | 1.33 (1.17-1.53) |
| *P* for trend |  | 0.01 |  |  | | <0.01 |  | |  | <0.01 |  |  | <0.01 |
| **TC (mmol/L)** |  |  |  |  | |  |  | |  |  |  |  |  |
| Above desirable (<5.2) | Reference | Reference |  | Reference | | Reference |  | | Reference | Reference |  | Reference | Reference |
| Borderline high (5.2-6.1) | 1.64 (1.48-1.82) | 1.45 (1.27-1.64) |  | 1.47 (1.32-1.64) | | 1.17 (1.02-1.34) |  | | 1.42 (1.26-1.59) | 1.08 (0.94-1.24) |  | 1.69 (1.52-1.88) | 1.40 (1.22-1.60) |
| High (≥6.2） | 1.99 (1.65-2.40) | 1.60 (1.24-2.06) |  | 1.82 (1.49-2.21) | | 1.31 (0.99-1.72) |  | | 1.77 (1.45-2.17) | 1.18 (0.89-1.55) |  | 2.49 (2.02-3.07) | 1.97 (1.49-2.62) |
| *P* for trend |  | <0.01 |  |  | | 0.01 |  | |  | 0.19 |  |  | <0.01 |
| **LDL-C (mmol/L)** |  |  |  |  | |  |  | |  |  |  |  |  |
| Desirable (<2.6) | Reference | Reference |  | Reference | | Reference |  | | Reference | Reference |  | Reference | Reference |
| Above desirable (2.6-3.3) | 1.34 (1.22-1.46) | 1.10 (0.99-1.22) |  | 1.35 (1.22-1.50) | | 1.16 (1.03-1.29) |  | | 1.39 (1.25-1.54) | 1.23 (1.09-1.38) |  | 1.45 (1.32-1.58) | 1.18 (1.06-1.30) |
| Borderline high (3.4-4.0) | 1.74 (1.49-2.02) | 1.12 (0.92-1.35) |  | 1.67 (1.42-1.97) | | 1.22 (0.98-1.51) |  | | 1.92 (1.63-2.27) | 1.59 (1.29-1.97) |  | 1.91 (1.62-2.24) | 1.16 (0.94-1.43) |
| High (≥4.1) | 2.02 (1.52-2.68) | 1.13 (0.79-1.61) |  | 1.71 (1.26-2.33) | | 1.11 (0.76-1.63) |  | | 1.98 (1.46-2.69) | 1.46 (0.98-2.17) |  | 2.26 (1.65-3.09) | 1.02 (0.68-1.53) |
| *P* for trend |  | 0.10 |  |  | | 0.03 |  | |  | <0.01 |  |  | 0.02 |
| **HDL-C (mmol/L)** |  |  |  |  | |  |  | |  |  |  |  |  |
| Normal (≥1.0) | Reference | Reference |  | Reference | | Reference |  | | Reference | Reference |  | Reference | Reference |
| Low (<1.0) | 1.05 (0.89-1.25) | 0.96 (0.80-1.16) |  | 1.21 (1.01-1.46) | | 1.09 (0.90-1.33) |  | | 0.81 (0.67-1.01) | 0.89 (0.72-1.11) |  | 1.03 (0.87-1.23) | 0.96 (0.79-1.15) |
| *P* for trend |  | 0.67 |  |  | | 0.38 |  | |  | 0.30 |  |  | 0.64 |
| **TG (mmol/L)** |  |  |  |  | |  |  | |  |  |  |  |  |
| Above desirable (<1.7) | Reference | Reference |  | Reference | | Reference |  | | Reference | Reference |  | Reference | Reference |
| Borderline high (1.7-2.2) | 1.35 (1.21-1.50) | 1.19 (1.06-1.34) |  | 1.75 (1.55-1.96) | | 1.56 (1.39-1.77) |  | | 1.14 (1.01-1.30) | 1.02 (0.90-1.17) |  | 1.53 (1.37-1.72) | 1.36 (1.21-1.53) |
| High (≥2.3) | 1.48 (1.31-1.66) | 1.19 (1.05-1.35) |  | 1.89 (1.68-2.14) | | 1.61 (1.41-1.84) |  | | 1.14 (1.00-1.30) | 1.04 (0.90-1.19) |  | 1.65 (1.46-1.86) | 1.35 (1.19-1.54) |
| *P* for trend |  | <0.01 |  |  | | <0.01 |  | |  | 0.65 |  |  | <0.01 |
| **Anthropometric characteristics ^c^** |  |  |  |  | |  |  | |  |  |  |  |  |
| **WC (cm)** |  |  |  |  | |  |  | |  |  |  |  |  |
| Normal | Reference | Reference |  | Reference | | Reference |  | | Reference | Reference |  | Reference | Reference |
| Non-standard | 1.21 (1.11-1.32) | 1.18 (1.07-1.29) |  | 1.65 (1.49-1.82) | | 1.41 (1.27-1.56) |  | | 1.22 (1.11-1.34) | 1.08 (0.97-1.20) |  | 1.40 (1.28-1.52) | 1.28 (1.16-1.40) |
| *P* for trend |  | <0.01 |  |  | | <0.01 |  | |  | 0.15 |  |  | <0.01 |
| **WHtR** |  |  |  |  | |  |  | |  |  |  |  |  |
| <0.5 | Reference | Reference |  | Reference | | Reference |  | | Reference | Reference |  | Reference | Reference |
| ≥0.5 | 1.43 (1.31-1.57) | 1.26 (1.15-1.40) |  | 1.71 (1.54-1.90) | | 1.41 (1.27-1.58) |  | | 1.13 (1.02-1.25) | 0.96 (0.86-1.07) |  | 1.53 (1.40-1.68) | 1.29 (1.17-1.42) |
| *P* for trend |  | <0.01 |  |  | | <0.01 |  | |  | 0.43 |  |  | <0.01 |
| **WHR** |  |  |  |  | |  |  | |  |  |  |  |  |
| Normal | Reference | Reference |  | Reference | | Reference |  | | Reference | Reference |  | Reference | Reference |
| Non-standard | 1.33 (1.22-1.45) | 1.21 (1.10-1.32) |  | 1.62 (1.47-1.78) | | 1.34 (1.21-1.49) |  | | 1.24 (1.12-1.37) | 1.08 (0.97-1.20) |  | 1.42 (1.31-1.55) | 1.23 (1.12-1.35) |
| *P* for trend |  | <0.01 |  |  | | <0.01 |  | |  | 0.14 |  |  | <0.01 |
| **BMI (kg/m^2^)** |  |  |  |  | |  |  | |  |  |  |  |  |
| Underweight (<18.5) | 0.58 (0.39-0.85) | 0.66 (0.45-0.98) |  | 0.62 (0.38-0.99) | | 0.70 (0.42-1.15) |  | | 0.94 (0.64-1.39) | 1.06 (0.71-1.57) |  | 0.75 (0.54-1.03) | 0.88 (0.63-1.22) |
| Normal (18.5-23.9) | Reference | Reference |  | Reference | | Reference |  | | Reference | Reference |  | Reference | Reference |
| Overweight (24.0-27.9) | 1.40 (1.27-1.54) | 1.27 (1.15-1.41) |  | 1.43 (1.28-1.60) | | 1.27 (1.13-1.42) |  | | 1.23 (1.10-1.39) | 1.18 (1.04-1.33) |  | 1.44 (1.31-1.59) | 1.30 (1.17-1.44) |
| Obesity (≥28.0) | 1.86 (1.63-2.11) | 1.60 (1.39-1.83) |  | 2.41 (2.09-2.76) | | 2.01 (1.73-2.33) |  | | 1.58 (1.37-1.83) | 1.47 (1.26-1.72) |  | 2.08 (1.82-2.38) | 1.75 (1.52-2.02) |
| *P* for trend |  | <0.01 |  |  | | <0.01 |  | |  | <0.01 |  |  | <0.01 |
| **PI (kg/m^3^)** |  |  |  |  | |  |  | |  |  |  |  |  |
| Q1 (<14.12) | Reference | Reference |  | Reference | | Reference |  | | Reference | Reference |  | Reference | Reference |
| Q2 (14.12-15.21) | 1.34 (1.19-1.51) | 1.28 (1.13-1.45) |  | 1.37 (1.19-1.59) | | 1.24 (1.07-1.43) |  | | 0.99 (0.87-1.14) | 0.95 (0.82-1.09) |  | 1.26 (1.12-1.42) | 1.18 (1.04-1.33) |
| Q3 (15.52-17.05) | 1.49 (1.31-1.69) | 1.42 (1.25-1.62) |  | 1.79 (1.55-2.08) | | 1.49 (1.28-1.73) |  | | 1.31 (1.13-1.51) | 1.19 (1.02-1.38) |  | 1.61 (1.43-1.83) | 1.45 (1.27-1.65) |
| Q4 (≥17.06) | 1.93 (1.70-2.19) | 1.83 (1.60-2.11) |  | 2.75 (2.38-3.19) | | 2.14 (1.83-2.50) |  | | 1.50 (1.30-1.73) | 1.31 (1.12-1.53) |  | 2.17 (1.90-2.47) | 1.88 (1.64-2.17) |
| *P* for trend |  | <0.01 |  |  | | <0.01 |  | |  | <0.01 |  |  | <0.01 |
| **CI (m^3/2^·kg^1/2^)** |  |  |  |  | |  |  | |  |  |  |  |  |
| Q1 (<43.10) | Reference | Reference |  | Reference | | Reference |  | | Reference | Reference |  | Reference | Reference |
| Q2 (43.10-48.49) | 1.36 (1.20-1.53) | 1.23 (1.08-1.39) |  | 1.31 (1.14-1.50) | | 1.20 (1.03-1.38) |  | | 1.06 (0.93-1.22) | 0.98 (0.85-1.13) |  | 1.36 (1.22-1.53) | 1.22 (1.08-1.37) |
| Q3 (48.50 -54.49) | 1.69 (1.49-1.92) | 1.40 (1.22-1.59) |  | 1.67 (1.45-1.93) | | 1.45 (1.24-1.68) |  | | 1.12 (0.97-1.29) | 1.00 (0.86-1.16) |  | 1.72 (1.53-1.95) | 1.41 (1.24-1.61) |
| Q4 (≥54.50) | 2.04 (1.79-2.33) | 1.53 (1.33-1.77) |  | 2.17 (1.87-2.50) | | 1.85 (1.58-2.17) |  | | 1.37 (1.18-1.58) | 1.18 (1.01-1.38) |  | 2.21 (1.94-2.52) | 1.68 (1.45-1.93) |
| *P* for trend |  | <0.01 |  |  | | <0.01 |  | |  | 0.04 |  |  | <0.01 |
| **RFM** |  |  |  |  | |  |  | |  |  |  |  |  |
| Q1 (<27.04) | Reference | Reference |  | Reference | | Reference |  | | Reference | Reference |  | Reference | Reference |
| Q2 (27.04-34.17) | 0.85 (0.75-0.97) | 1.08 (0.94-1.25) |  | 1.27 (1.11-1.47) | | 1.38 (1.18-1.63) |  | | 0.97 (0.84-1.11) | 0.95 (0.81-1.12) |  | 0.96 (0.85-1.08) | 1.17 (1.01-1.36) |
| Q3 (34.18-39.00) | 0.71 (0.62-0.80) | 1.28 (1.04-1.58) |  | 1.38 (1.20-1.60) | | 1.79 (1.42-2.25) |  | | 1.00 (0.86-1.16) | 0.93 (0.74-1.17) |  | 0.89 (0.79-1.01) | 1.38 (1.13-1.68) |
| Q4 (≥39.00) | 0.97 (0.86-1.10) | 1.67 (1.35-2.08) |  | 2.00 (1.73-2.31) | | 2.31 (1.82-2.94) |  | | 1.19 (1.03-1.38) | 0.97 (0.76-1.23) |  | 1.24 (1.09-1.42) | 1.74 (1.40-2.14) |
| *P* for trend |  | <0.01 |  |  | | <0.01 |  | |  | 0.96 |  |  | <0.01 |
| **AVI (L)** |  |  |  |  | |  |  | |  |  |  |  |  |
| Q1 (<12.21) | Reference | Reference |  | Reference | | Reference |  | | Reference | Reference |  | Reference | Reference |
| Q2 (12.21-14.19) | 1.23 (1.10-1.39) | 1.11 (0.98-1.26) |  | 1.29 (1.13-1.48) | | 1.16 (1.01-1.33) |  | | 0.96 (0.84-1.09) | 0.86 (0.75-0.99) |  | 1.24 (1.11-1.39) | 1.10 (0.98-1.24) |
| Q3 (14.20-16.40) | 1.53 (1.36-1.73) | 1.27 (1.12-1.44) |  | 1.56 (1.36-1.79) | | 1.33 (1.15-1.54) |  | | 1.07 (0.93-1.22) | 0.92 (0.79-1.06) |  | 1.55 (1.38-1.75) | 1.26 (1.11-1.43) |
| Q4 (≥16.40) | 1.86 (1.64-2.11) | 1.40 (1.22-1.61) |  | 2.01 (1.75-2.31) | | 1.67 (1.43-1.94) |  | | 1.22 (1.06-1.40) | 0.99 (0.85-1.15) |  | 2.00 (1.76-2.27) | 1.49 (1.30-1.71) |
| *P* for trend |  | <0.01 |  |  | | <0.01 |  | |  | 0.87 |  |  | <0.01 |
| **LAP (cm·mmol/L)** |  |  |  |  | |  |  | |  |  |  |  |  |
| Q1 (<17.81) | Reference | Reference |  | Reference | | Reference |  | | Reference | Reference |  | Reference | Reference |
| Q2 (17.81-30.74) | 1.19 (1.06-1.34) | 1.12 (0.99-1.26) |  | 1.37 (1.19-1.58) | | 1.27 (1.10-1.47) |  | | 1.04 (0.91-1.19) | 0.94 (0.82-1.08) |  | 1.22 (1.09-1.36) | 1.13 (1.00-1.26) |
| Q3 (30.75-52.13) | 1.45 (1.28-1.63) | 1.28 (1.12-1.47) |  | 2.11 (1.84-2.42) | | 1.80 (1.55-2.10) |  | | 1.24 (1.08-1.42) | 1.05 (0.90-1.22) |  | 1.70 (1.51-1.91) | 1.45 (1.27-1.65) |
| Q4 (≥52.14) | 1.71 (1.52-1.94) | 1.35 (1.11-1.63) |  | 3.01 (2.60-3.47) | | 2.43 (1.98-2.98) |  | | 1.32 (1.14-1.52) | 1.12 (0.91-1.39) |  | 2.14 (1.89-2.43) | 1.64 (1.36-1.99) |
| *P* for trend |  | <0.01 |  |  | | <0.01 |  | |  | 0.27 |  |  | <0.01 |
| **VAI** |  |  |  |  | |  |  | |  |  |  |  |  |
| Q1 (<0.95) | Reference | Reference |  | Reference | | Reference |  | | Reference | Reference |  | Reference | Reference |
| Q2 (0.95-1.46) | 0.90 (0.81-1.01) | 0.84 (0.27-2.55) |  | 1.32 (1.15-1.51) | | 1.32 (0.49-3.51) |  | | 1.12 (0.98-1.28) | 0.81 (0.27-2.38) |  | 1.03 (0.92-1.16) | 0.84 (0.32-2.20) |
| Q3 (1.47-2.35) | 0.96 (0.86-1.08) | 1.03 (0.32-3.31) |  | 1.81 (1.58-2.07) | | 1.30 (0.31-5.57) |  | | 1.30 (1.14-1.49) | 0.96 (0.19-4.81) |  | 1.25 (1.12-1.41) | 1.20 (0.32-4.56) |
| Q4 (≥2.36) | 1.16 (1.03-1.31) | 1.35 (0.82-2.21) |  | 2.46 (2.14-2.83) | | 1.07 (0.66-1.75) |  | | 1.24 (1.07-1.43) | 1.29 (0.74-2.25) |  | 1.52 (1.34-1.72) | 1.48 (0.86-2.56) |
| *P* for trend |  | 0.26 |  |  | | 0.70 |  | |  | 0.40 |  |  | 0.17 |
| **CVAI** |  |  |  |  | |  |  | |  |  |  |  |  |
| Q1 (<66.50) | Reference | Reference |  | Reference | | Reference |  | | Reference | Reference |  | Reference | Reference |
| Q2 (66.50-92.09) | 1.21 (1.08-1.37) | 1.10 (0.97-1.25) |  | 1.54 (1.32-1.78) | | 1.32 (1.13-1.54) |  | | 1.37 (1.19-1.59) | 1.13 (0.97-1.31) |  | 1.46 (1.30-1.64) | 1.25 (1.11-1.42) |
| Q3 (92.10-115.59) | 1.38 (1.22-1.56) | 1.14 (0.99-1.30) |  | 2.22 (1.91-2.57) | | 1.70 (1.45-2.00) |  | | 1.62 (1.40-1.88) | 1.19 (1.01-1.40) |  | 1.83 (1.62-2.06) | 1.37 (1.20-1.57) |
| Q4 (≥115.60) | 1.86 (1.64-2.11) | 1.39 (1.20-1.62) |  | 3.20 (2.76-3.71) | | 2.26 (1.90-2.70) |  | | 1.98 (1.71-2.30) | 1.41 (1.18-1.67) |  | 2.65 (2.33-3.03) | 1.80 (1.54-2.10) |
| *P* for trend |  | <0.01 |  |  | | <0.01 |  | |  | <0.01 |  |  | <0.01 |
| **BRI** |  |  |  |  | |  |  | |  |  |  |  |  |
| Q1 (<3.12) | Reference | Reference |  | Reference | | Reference |  | | Reference | Reference |  | Reference | Reference |
| Q2 (3.12-3.84) | 1.23 (1.09-1.39) | 1.14 (1.01-1.29) |  | 1.40 (1.22-1.59) | | 1.25 (1.09-1.43) |  | | 1.07 (0.94-1.22) | 0.96 (0.84-1.10) |  | 1.29 (1.15-1.44) | 1.15 (1.02-1.29) |
| Q3 (3.85-4.60) | 1.53 (1.36-1.73) | 1.33 (1.17-1.51) |  | 1.78 (1.55-2.05) | | 1.45 (1.25-1.67) |  | | 1.17 (1.02-1.34) | 0.98 (0.85-1.13) |  | 1.66 (1.48-1.88) | 1.37 (1.21-1.56) |
| Q4 (≥4.61) | 1.69 (1.49-1.92) | 1.44 (1.26-1.65) |  | 2.48 (2.15-2.85) | | 1.87 (1.61-2.17) |  | | 1.34 (1.17-1.55) | 1.04 (0.90-1.21) |  | 1.97 (1.73-2.23) | 1.52 (1.33-1.74) |
| *P* for trend |  | <0.01 |  |  | | <0.01 |  | |  | 0.50 |  |  | <0.01 |
| **BAE** |  |  |  |  | |  |  | |  |  |  |  |  |
| Q1 (<26.88) | Reference | Reference |  | Reference | | Reference |  | | Reference | Reference |  | Reference | Reference |
| Q2 (26.88-33.45) | 0.93 (0.82-1.06) | 1.27 (1.09-1.47) |  | 1.38 (1.19-1.61) | | 1.57 (1.32-1.87) |  | | 1.14 (0.98-1.33) | 1.28 (1.08-1.53) |  | 1.06 (0.93-1.20) | 1.45 (1.24-1.70) |
| Q3 (33.46-38.16) | 0.75 (0.66-0.85) | 1.44 (1.18-1.75) |  | 1.48 (1.27-1.73) | | 2.04 (1.63-2.55) |  | | 1.27 (1.09-1.48) | 1.62 (1.29-2.04) |  | 1.01 (0.89-1.15) | 1.78 (1.46-2.18) |
| Q4 (≥38.17) | 1.12 (0.99-1.28) | 2.12 (1.72-2.62) |  | 2.51 (2.17-2.92) | | 3.17 (2.50-4.03) |  | | 1.62 (1.39-1.88) | 1.87 (1.47-2.40) |  | 1.56 (1.37-1.78) | 2.56 (2.06-3.17) |
| *P* for trend |  | <0.01 |  |  | | <0.01 |  | |  | <0.01 |  |  | <0.01 |
| **Anthropometric prediction equation ^c, d^** |  |  |  |  | |  |  | |  |  |  |  |  |
| **Lean body mass (kg)** |  |  |  |  | |  |  | |  |  |  |  |  |
| Q1 (<34.70) | Reference | Reference |  | Reference | | Reference |  | | Reference | Reference |  | Reference | Reference |
| Q2 (34.70-38.79) | 1.16 (1.02-1.32) | 1.05 (0.90-1.24) |  | 1.13 (0.98-1.29) | | 1.02 (0.85-1.21) |  | | 1.12 (0.97-1.30) | 1.12 (0.94-1.35) |  | 1.15 (1.02-1.31) | 1.04 (0.88-1.21) |
| Q3 (38.80-47.89) | 1.51 (1.32-1.73) | 1.33 (1.06-1.67) |  | 1.13 (0.98-1.31) | | 1.24 (0.97-1.58) |  | | 1.15 (0.99-1.34) | 1.31 (1.01-1.70) |  | 1.42 (1.24-1.62) | 1.27 (1.00-1.60) |
| Q4 (≥47.90) | 1.95 (1.71-2.23) | 1.58 (1.16-2.17) |  | 1.11 (0.95-1.28) | | 1.53 (1.09-2.16) |  | | 1.17 (1.01-1.38) | 1.66 (1.17-2.37) |  | 1.65 (1.44-1.89) | 1.51 (1.09-2.10) |
| *P* for trend |  | <0.01 |  |  | | 0.02 |  | |  | 0.01 |  |  | 0.02 |
| **Fat mass (kg)** |  |  |  |  | |  |  | |  |  |  |  |  |
| Q1 (<17.80) | Reference | Reference |  | Reference | | Reference |  | | Reference | Reference |  | Reference | Reference |
| Q2 (17.80-21.75) | 1.00 (0.88-1.13) | 1.08 (0.94-1.24) |  | 1.35 (1.17-1.56) | | 1.27 (1.08-1.49) |  | | 1.00 (0.87-1.15) | 0.94 (0.80-1.11) |  | 1.08 (0.96-1.23) | 1.12 (0.97-1.29) |
| Q3 (21.76-25.70) | 1.15 (1.02-1.31) | 1.32 (1.12-1.54) |  | 1.68 (1.45-1.95) | | 1.54 (1.29-1.84) |  | | 1.20 (1.04-1.39) | 1.09 (0.91-1.30) |  | 1.29 (1.14-1.47) | 1.37 (1.17-1.61) |
| Q4 (≥25.71) | 1.34 (1.18-1.52) | 1.44 (1.19-1.75) |  | 2.40 (2.07-2.78) | | 1.98 (1.60-2.45) |  | | 1.44 (1.24-1.66) | 1.16 (0.93-1.44) |  | 1.72 (1.51-1.96) | 1.67 (1.37-2.03) |
| *P* for trend |  | <0.01 |  |  | | <0.01 |  | |  | 0.11 |  |  | <0.01 |
| **Percent fat (%)** |  |  |  |  | |  |  | |  |  |  |  |  |
| Q1 (<27.30) | Reference | Reference |  | Reference | | Reference |  | | Reference | Reference |  | Reference | Reference |
| Q2 (27.30-35.99) | 0.78 (0.68-0.88) | 1.11 (0.95-1.29) |  | 1.13 (0.97-1.31) | | 1.39 (1.17-1.64) |  | | 0.80 (0.69-0.93) | 0.91 (0.76-1.08) |  | 0.84 (0.74-0.95) | 1.22 (1.03-1.43) |
| Q3 (36.00-39.29) | 0.66 (0.58-0.75) | 1.39 (1.11-1.74) |  | 1.22 (1.05-1.42) | | 1.85 (1.43-2.40) |  | | 0.95 (0.82-1.10) | 1.07 (0.83-1.38) |  | 0.83 (0.72-0.94) | 1.56 (1.25-1.95) |
| Q4 (≥39.30) | 0.97 (0.86-1.11) | 1.93 (1.52-2.43) |  | 2.14 (1.85-2.47) | | 2.84 (2.18-3.70) |  | | 1.29 (1.12-1.50) | 1.27 (0.97-1.65) |  | 1.32 (1.15-1.51) | 2.21 (1.75-2.80) |
| *P* for trend |  | <0.01 |  |  | | <0.01 |  | |  | 0.01 |  |  | <0.01 |
| *aOR: adjusted OR; Results are based on a generalized estimating equation (GEE) with prediabetes (IFG, IGT, risk HbA1c) *vs*. normal group as binary outcome (analysis sample n=9779). | | | | | | | | | | | | | |
| y, years; n, number; IFG: impaired fasting glucose; IGT: impaired glucose tolerance; HbA1c: glycosylated hemoglobin; Q: quartile; TC: total cholesterol; LDL-C: low-density lipoprotein cholesterol; HDL-C: high-density lipoprotein cholesterol; TG: triglyceride; WC: waist circumference; WHtR: waist-to-height ratio; WHR: waist-to-hip ratio; BMI: body mass index; PI: ponderal index; CI: conicity index; RFM: relative fat mass; AVI: abdominal volume index; LAP: lipid accumulation product; VAI: visceral adiposity index; CVAI: Chinese visceral adiposity index; BRI: body roundness index; BAE: body adiposity estimator. | | | | | | | | | | | | | |
| a. Prediabetes: IFG or IGT or elevated HbA1c; | | | | | | | | | | | | | |
| b. For demographic characteristics, behavioral characteristics and clinical and biochemical characteristics, multivariable-adjusted OR (95% CI) were adjusted for age, gender, education level, equivalent household income, drug history, family history of diabetes, smoking status, drinking status, regular exercise, hypertension, TC, LDL-C, HDL-C, TG, BMI; | | | | | | | | | | | | | |
| c. For anthropometric characteristics and anthropometric prediction equation, adjusted OR (95% CI) were additionally adjusted for age, gender, education level, equivalent household income, drug history, family history of diabetes, smoking status, drinking status, regular exercise, hypertension, TC, LDL-C, HDL-C, TG; Additionally, fat mass and height were further adjusted for lean body mass, lean body mass and height were further adjusted for fat mass; | | | | | | | | | | | | | |
| d. Lean body mass, fat mass and percent fat were derived from a validated anthropometric prediction equation. | | | | | | | | | | | | | |
